# A real-time driver fatigue identification method based on GA-GRNN

**DOI:** 10.3389/fpubh.2022.991350

**Published:** 2022-10-20

**Authors:** Xiaoyuan Wang, Longfei Chen, Yang Zhang, Huili Shi, Gang Wang, Quanzheng Wang, Junyan Han, Fusheng Zhong

**Affiliations:** ^1^College of Electromechanical Engineering, Qingdao University of Science and Technology, Qingdao, China; ^2^Collaborative Innovation Center for Intelligent Green Manufacturing Technology and Equipment of Shandong, Qingdao, China

**Keywords:** fatigue driving, active safety warning system, machine vision, generalization regression neural network, genetic algorithm

## Abstract

It is of great practical and theoretical significance to identify driver fatigue state in real time and accurately and provide active safety warning in time. In this paper, a non-invasive and low-cost method of fatigue driving state identification based on genetic algorithm optimization of generalized regression neural network model is proposed. The specific work is as follows: (1) design simulated driving experiment and real driving experiment, determine the fatigue state of drivers according to the binary Karolinska Sleepiness Scale (KSS), and establish the fatigue driving sample database. (2) Improved Multi-Task Cascaded Convolutional Networks (MTCNN) and applied to face detection. Dlib library was used to extract the coordinate values of face feature points, collect the characteristic parameters of driver's eyes and mouth, and calculate the Euler Angle parameters of head posture. A fatigue identification model was constructed by using multiple characteristic parameters. (3) Genetic Algorithm (GA) was used to find the optimal smooth factor of Generalized Regression Neural Network (GRNN) and construct GA-GRNN fatigue driving identification model. Compared with K-Nearest Neighbor (KNN), Random Forest (RF), and GRNN fatigue driving identification algorithms. GA-GRNN has the best generalization ability and high stability, with an accuracy of 93.3%. This study provides theoretical and technical support for the application of driver fatigue identification.

## Introduction

Fatigue driving is often accompanied by drowsiness or decreased physical function. Drivers who continue to drive under fatigue conditions are prone to road traffic accidents. Fatigue is defined as a complex state, a transitional state from wakefulness to sleep. However, there is still no unified definition of fatigue driving (1). In our study, fatigue driving is defined as the driver's decrease in alertness, mental function, and physiological factors (2).

**Figure 1:**
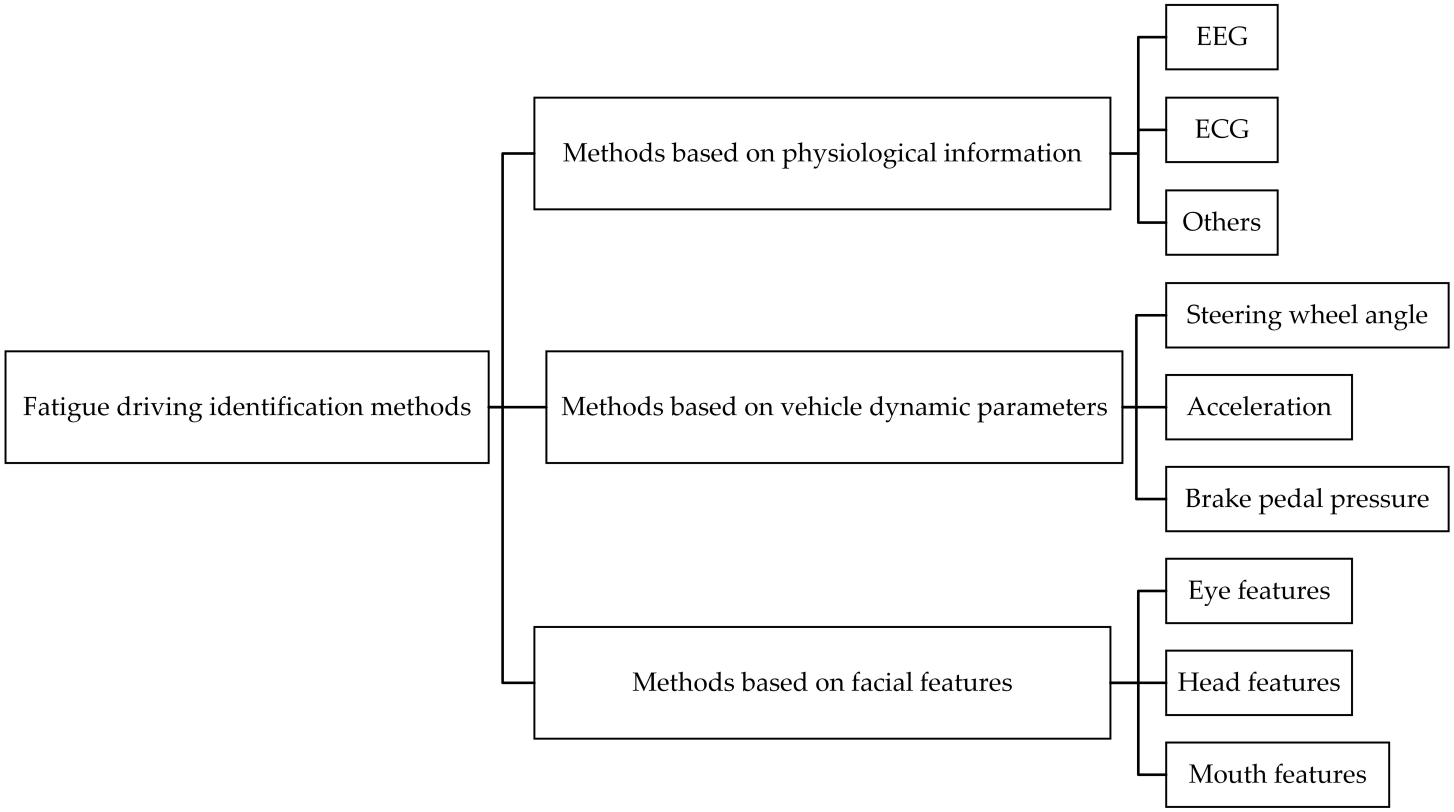
The classification of the fatigue driving identification methods.

Fatigue driving identification is mainly divided into two methods: the subjective assessment method and the objective detection method (3). Driver fatigue can be quantified with the subjective assessment method through offline questionnaires, including KSS (4), Pearson Sleepiness Scale (PSS), and the Stanford Sleepiness Scale (SSS). Auxiliary tools or sensors are used in the objective detection method to detect the driver's physiological information (5–9), vehicle dynamic parameters, and facial features, which can determine whether the driver is in a fatigued state. The facial features of driver including eyes, mouth, head and expressions can be extracted in the fatigue identification method based on facial features. The classification of the methods is shown in Figure 1.

The fatigue driving identification method based on physiological information usually requires the driver to wear a variety of sensors or monitoring equipment, which is easy to cause the driver to feel disgusted and uncomfortable. The cost of human equipment is high, which is difficult to promote and apply in driving assistance systems. The method based on vehicle parameters is an indirect detection method, which cannot intuitively express the driver's fatigue state. Compared with the other two identification methods, this method has the advantages of non-invasiveness, not affected by the driver's driving habits, and good real-time performance.

Savas et al. (10) extracted the percentage of eyelid closure (PERCLOS) and yawning frequency as feature parameters to recognition the fatigue driving state. Berkati et al. (11) extracted the driver's blink rate, blink time, PERCLOS, and other characteristic parameters. They constructed an RBF (Radial Basis Function) neural network to identify the fatigue driving state. Phan et al. (12) used the SSD-ResNet-10 model for face detection and location. They pre-trained the adaptive ResNet-50V2 neural network recognition model on the RMFD (Real-World Masked Face Dataset) through transfer learning. The self-built fatigue datasets was used to fine-tune the identified model parameters to achieve higher model accuracy with 97.3%. Cheng et al. (13) extracted EAR (eye aspect ratio) and MAR (mouth aspect ratio) parameters. The average accuracy of the trained logistic regression model for fatigue driving identification was 83.7%. You et al. (14) trained a SVM (Support Vector Machine) model with the vertical and horizontal EAR features to identify the fatigue driving state. The proposed algorithm had an accuracy of 94.8% for the fatigue driving identification. Li et al. (15) preprocessed the face image through grayscale processing and gamma correction. The Dlib face alignment model was used to intercept the driver's eyes and mouth areas, the LBP (Local Binary Patterns) algorithm was used to extract feature vectors in the three areas. The model was trained with the radial basis SVM model and achieved an accuracy of 96.07%. Zhuang et al. (16) used Dlib face key points to segment the eye image. A lightweight U-Net network was used to perform pixel-level classification of the eye image, which could accurately extract the driver's pupil and iris features. After training the eye characteristic with the decision network, the fatigue driving recognition was finally realized based on the PERCLOS criterion. Jia et al. (17) fused the parameters of ECR (eye closure rate), MOR (mouth opening rate), and HNFR (head non-frontal rate) to identify fatigue driving state. They achieved a recognition rate of 97.5% on the self-made data set. Liu et al. (18) proposed an Adaboost algorithm based on multi-block LBP features to locate face feature points. The opening angles of the eye and mouth could be calculated through the coordinate values of the feature points on the image, and a fuzzy inference system was used to detect driver fatigue.

However, the methods based on facial features can be affected with the changing driving environment easily. It is difficult to import it into a small-embedded system with the large-scale model. The single evaluation criterion PERCLOS or threshold method to judge fatigue driving has certain limitations. Most scholars use open source fatigue driving data sets or simulated driving experimental data sets to train the fatigue driving state identification model, and rarely consider combining the real vehicle driving experimental data and the simulated driving experimental data to train the identification model. The optimal parameter matrix utilization of the data is not high. A non-invasive and low-cost method of fatigue driving state identification based on genetic algorithm optimization of generalized regression neural network model is proposed in this paper, which uses the eye, mouth and head parameters. The driver's face area can be quickly detected and accurately located with the improved MTCNN. The dlib library can be used to locate the key points of the face, which can be applied to extract the parameters of the eye and mouth. The Euler angles of the head pose are calculated according to the corresponding relationship between the two-dimensional face key point coordinates and the three-dimensional face coordinate system. The raw data is processed by fast Fourier filtering algorithm and factor analysis, and the common factor is extracted as the characteristic parameter to identify the fatigue driving behavior. The processed feature parameters are input into the optimized generalized regression neural network identification model. Compared with KNN, RF, GRNN fatigue driving identification algorithms, the results show that the system has an accuracy rate of 93.3%, a recall rate of 91.4%, a precision rate of 92.9%, and the F1 score of 92.1%.

## Materials and methods

### Participants

33 participants with regular physiological routines participate in the experiment, including 21 males and 12 females. The participants of the experiment are all students and faculty members, and some of them wear glasses. The age distribution of the participants is between 21 and 52 years old (mean age 33.27 years, standard deviation 2.83 years). Driving experience is distributed between 1 and 13 years, with an average driving experience of 5.7 years. All participants must have at least 1 year of driving experience. Each participant is in good health, has regular diet and sleep, and has no bad habits. They will not be informed about the purpose of the experiment.

### Apparatus

**Figure 2:**
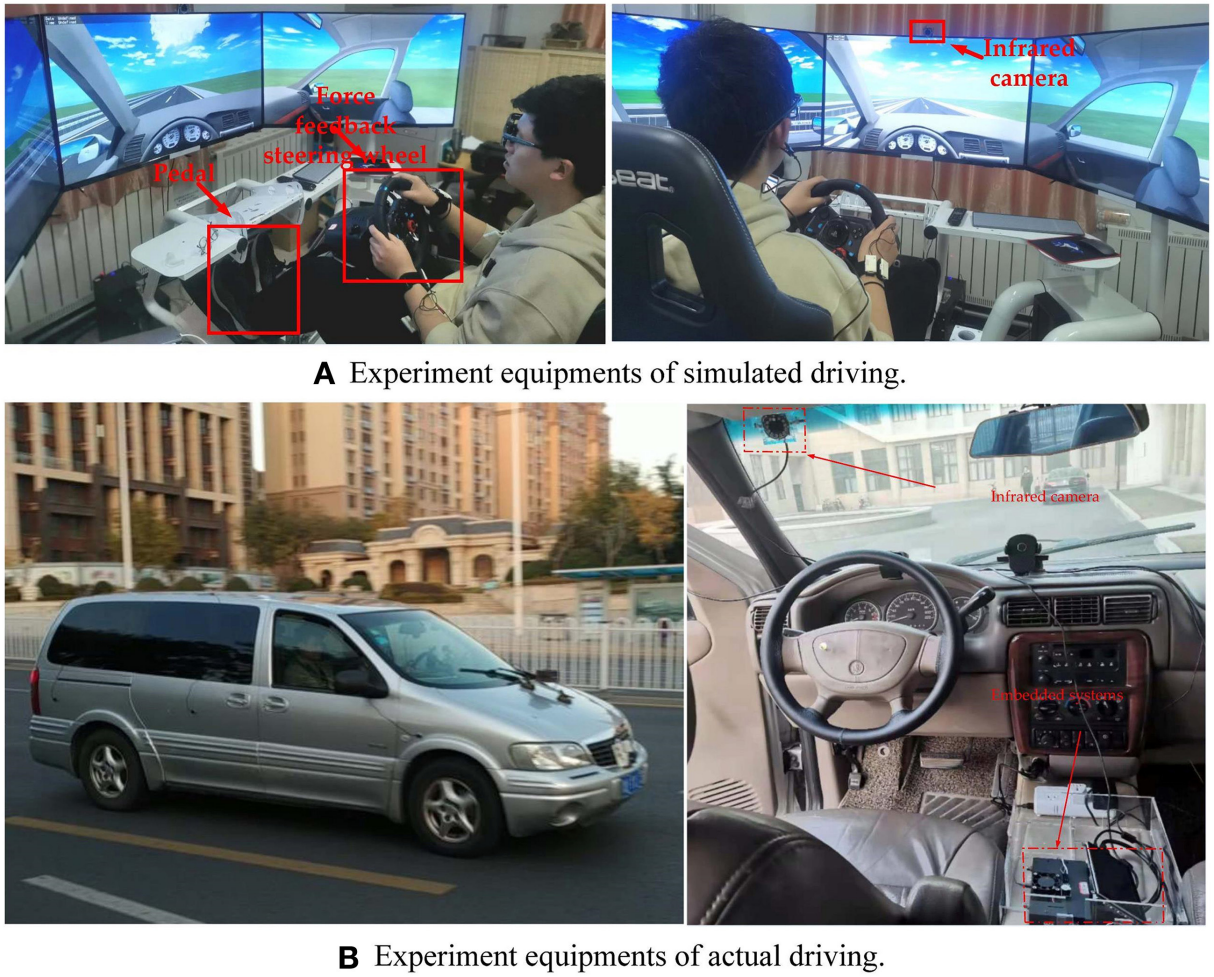
Experiment equipments of simulated driving **(A)** and actual driving **(B)**.

The experimental equipment used in this experiment includes driving simulation system, Buick GL8 experimental vehicle, infrared camera, Jetson Nano, and portable display screen. The G29 simulation driving kits produced by Logitech is selected in our study, including force feedback steering wheel, gear shift lever, adjustable brake pedal, accelerator, driving seat, etc. The Buick GL8 is used as the experimental car, and the infrared camera is fixed on the upper left of the front windshield of the car. The experimental equipment is shown in Figure 2.

### Procedure

**Figure 3:**
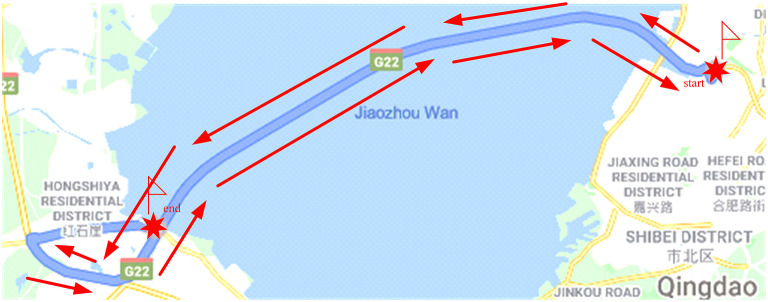
Experiment route for actual driving.

It is easy for the driver to enter a fatigued driving state on a highway with a monotonous driving environment (2). The road alignment changes are simple and the road environment lacks changes and stimulation on the visual sense. A monotonous driving environment is selected to carry out the real vehicle and simulated driving experiments in this paper. The driving route of the real vehicle experiment choose the section of Qingdao Jiaozhou Bay Cross Sea Bridge (from Sifang Campus of Qingdao University of Science and Technology to Huangdao East Toll Station). The total length of the experimental road section is 36 km, and the road speed limit is 80 km/h. Most of the road along the way is seawater, with a wide field of vision and a single road environment, which is no significant difference. The experimental route of the real vehicle is shown in Figure 3.

The experimental scene in simulated driving is written by UC-win/Road5.0 software. In order to ensure that the driving environment generated by the simulation software is similar to the real vehicle driving traffic environment, the simulated driving experimental scene in this paper adopts a circular two-way four-lane road, which consists of straight sections and multiple flat curve sections of different types. The total length of the road is about 53 km. The width of the single lane is about 3.75 meters. The road surface is flat and open, and there is no interference from pedestrians and other vehicles. In order to make the simulated driving scene more realistic, trees and grass are added on both sides of the road lane, and a separation zone is set in the center of the road. This road segment is repeated throughout the experiment until the end of the experiment.

The participants are required not to stay up late 1 day before the experiment to ensure adequate sleep. They cannot to take medicine, smoking, or drinking within 12 h before the experiment, and not to drink functional drinks or coffee within 3 h before the experiment. The time of 12:30–17:00 pm and 0:30–5:30 are selected for experiments. Considering the danger of the real car driving experiment, the real car experiment is only carried out in the time of 12:30–5:00 pm. Each participant needs to complete the driving of the experimental route. The infrared camera throughout the process can collect the facial video data of the participants.

In the simulated driving experiment, it is necessary to ensure that the participants must complete the two experiments in the afternoon and the early morning. If the two experiments cannot be completed at the same time, the video data of the tested driver will not be retained. The average driving time of the driver in the severe fatigue state in the simulated driving experiment is 3.9 h (19). Therefore, in this study, each participant conducted a simulated driving experiment for at least 4 h. The acquisition of video data is stopped 30 min after the participants has the signs of fatigue (20), and the experiment is ended. If the participant still has no signs of fatigue driving after 4 h of simulated driving experiment, the experiment will be terminated.

In order to avoid the invasiveness and the influence of individual subjective differences caused by the self-assessment method, the expert scoring method is adopted to determine the driver's fatigue level according to the KSS scale (4). Before the driving experiment, the experimental assistant installs, connects and debugs the experimental equipment. Participants should be trained before using the driving simulator and GL8 experimental vehicle and have sufficient time to practice and become familiar with them. During the experiment, the environment inside the car should be kept quiet, and there should be no noise in the car to disturb the driver. Three experts always pay attention to the mental state and external performance of the experimental subjects, evaluate the driver's fatigue state every 5 min (9), and mark on the scale until the end of the experiment. In this paper, when the KSS is < 3, it is considered as a non-fatigue driving state. When the KSS is >4, it is considered as a fatigue driving state (1).

After the experiment, the staff organizes the experimental instruments and related experimental equipment, and divides the video according to the KSS score and time interval during the experiment.

### Data collection algorithm

#### Improved MTCNN face detection algorithm

**Figure 4:**
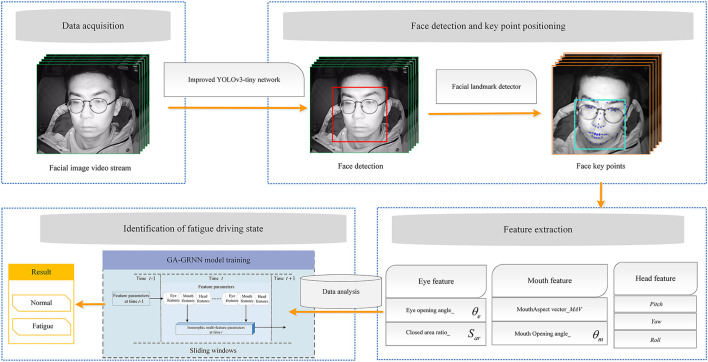
The algorithm structure diagram.

The improved MTCNN network is used for face detection and locates the driver's face area in this paper. The algorithm structure diagram is shown in Figure 4. The Dlib library is used to extract the coordinate values of face feature points, and extract the driver's eye, mouth and head feature parameters. Changes of characteristic parameters of blinking, eye opening degree, yawning, mouth opening degree, abnormal head posture and other phenomena in fatigue state are analyzed.

**Figure 5:**
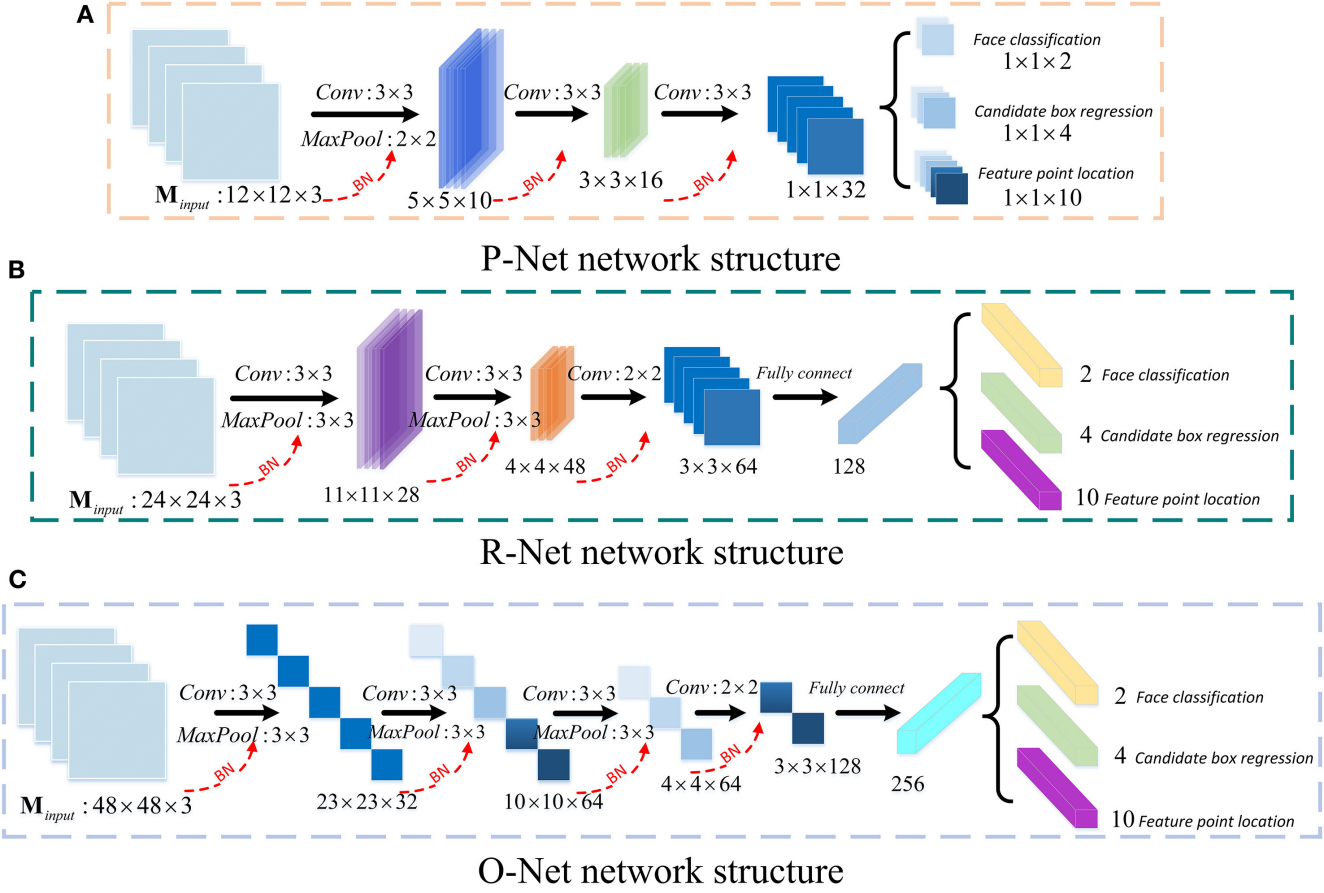
The structure of P-Net **(A)**, R-Net **(B)**, O-Net **(C)**.

MTCNN is a face detection model based on the face alignment method (21). Due to the slow convergence speed and the long training time of the original MTCNN model, it consumes too much computing resources. An improved MTCNN model is proposed for face detection in this paper. The batch normalization (BN) layer is added after the convolutional layer in the original MTCNN, and the face bounding box regression loss function is improved to speed up the convergence of the face detection model to obtain higher model detection accuracy. The network structures of the three sub-networks of MTCNN, P-Net, R-Net, and O-Net, are shown in Figure 5.

In the face detection box regression task, the offset between the candidate box and the ground-truth box is used for prediction. In this paper, the value is added to the loss function. The improved bounding box can be calculated as follows:


$$\begin{array}{l} {\text{L}}_{\text{i}}^{\text{box}}=1-IoU+\frac{\rho^{2}\left({\text{b}}^{\text{pb}},{\text{b}}^{\text{gt}}\right)}{{\text{c}}^{2}}+\frac{\frac{4}{\pi^{2}}{\left(\text{arctan}\frac{{\text{w}}^{\text{gt}}}{{\text{h}}^{\text{gt}}}-\text{arctan}\frac{{\text{w}}^{\text{pb}}}{{\text{h}}^{\text{pb}}}\right)}^{4}}{\left(1-IoU\right)}\\ \;+\frac{4}{\pi^{2}}{\left(\text{arctan}\frac{{\text{w}}^{\text{gt}}}{{\text{h}}^{\text{gt}}}-\text{arctan}\frac{{\text{w}}^{\text{pb}}}{{\text{h}}^{\text{pb}}}\right)}^{2} \end{array}$$

IoU is the intersection ratio between the predicted face frame and the real face frame. When IoU = 1, the predicted face frame overlaps with the real face frame. ρ^2^(*b*^*pb*^, *b*^*gt*^) is the square of the Euclidean distance between the predicted face frame and the real face frame. *c* is the diagonal length of the minimum bounding box formed by the predicted face frame *b*^*pb*^ and the real face frame *b*^*gt*^. *w*^*gt*^ and *h*^*gt*^ are the width and height of the real face frame, *w*^*pb*^ and *h*^*pb*^ are the width and height of the predicted face frame.

**Figure 6:**
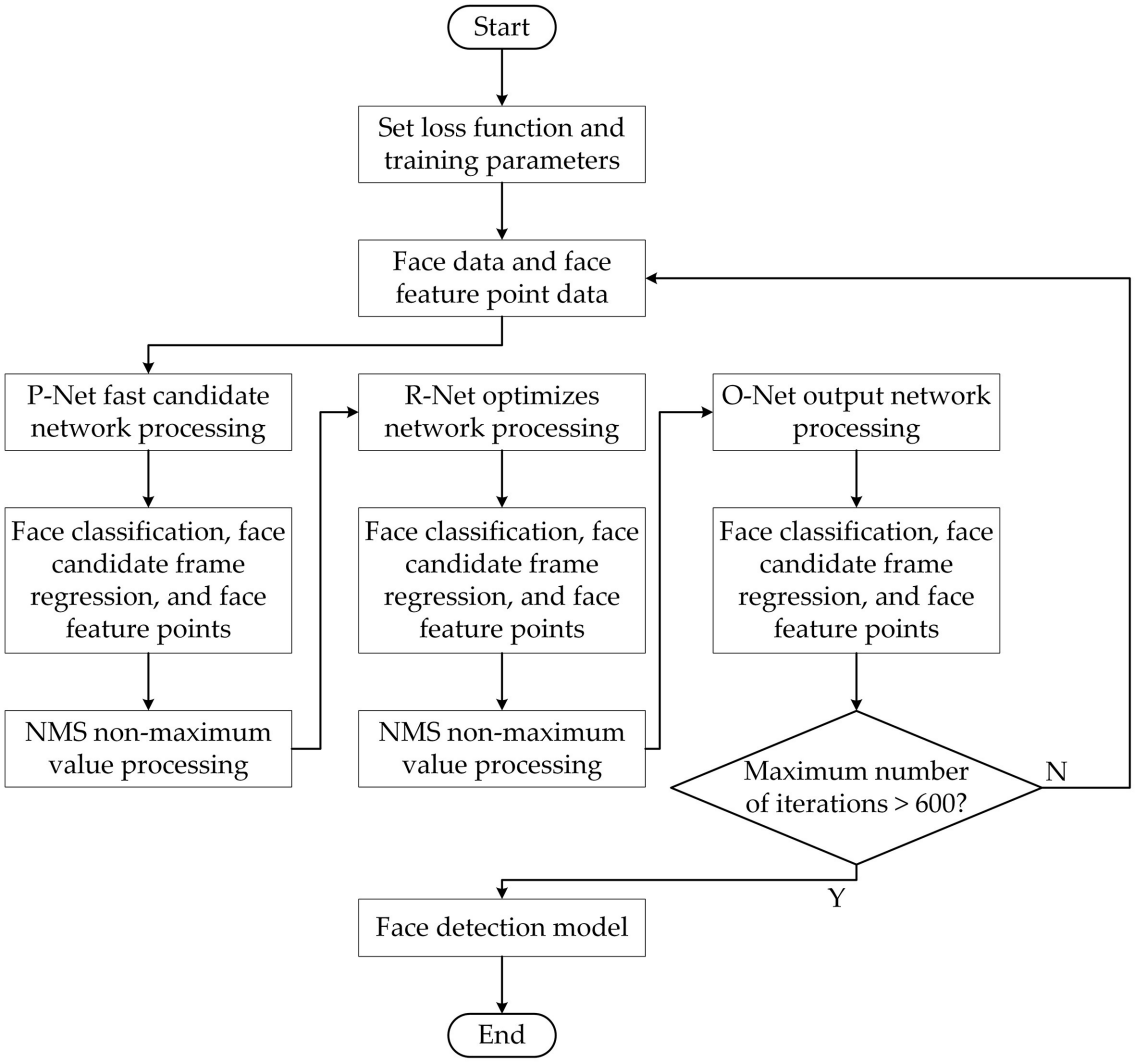
Training process of improved MTCNN.

The improved MTCNN model training process is shown in Figure 6. It can be seen from the figure that the face area detection frame can be quickly filtered by the P-Net network, and NMS (Non-Maximum Suppression) is used to eliminate the wrong redundant area frame. The face area is further processes with the O-Net network, and the face candidate frame is further screened by NMS. The final face detection area is filtered out through the O-Net network. The improved MTCNN face detection model starts to be trained until the data set used for training, the loss function and related training parameters are set.

**Figure 7:**
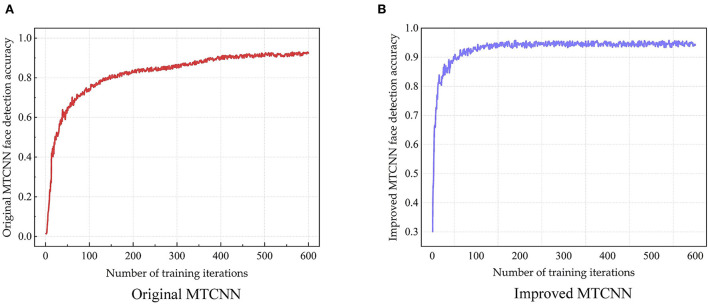
The training results of MTCNN face detection model—Accuracy of original MTCNN **(A)** and improved MTCNN **(B)**.

In order to verify the performance of the improved MTCNN face detection model, the MTCNN model before and after the improvement is compared on the Wider Face dataset and the DCNN (Dynamic Convolution Neural Network) face key point dataset. In order to reduce the influence of training parameters on the model training results, the parameters as other software and hardware, optimization algorithm, learning rate, and optimization function are same as before except the model. The training results are shown in Figure 7.

The training results of the model for 600 iterations can be seen from Figure 7. As shown in Figure 7A, the final face detection accuracy after the original MTCNN training is 0.938. As shown in Figure 7B, the final face detection accuracy of the improved MTCNN after training is 0.953. Compared with before optimization, the face detection accuracy of the improved MTCNN is improved by 1.5%.

The detection speed of the improved MTCNN face detection model proposed in this paper is faster than other models, and the average detection time per frame is 34 ms. Although it is not as accurate as the SSD (Single Shot MultiBox Detector) face detection model in the literature (22), the detection speed is greatly reduced when the SSD is transplanted into the Jetson Nano with limited computing power. It cannot meet the requirements of real-time identification of fatigue driving state, and it is difficult to apply in actual fatigue driving identification scenarios with high real-time requirements. The improved MTCNN face detection model proposed in this paper is more suitable for transplantation to embedded systems.

#### Face key point location based on Dlib

After locating the driver's face area, the driver's facial feature parameters in the face area will be extracted. The face alignment model based on the packaged Dlib library is adopted to locate the key points of the driver's face. The algorithm is less computationally time-consuming and more efficient, and can locate the key points of the face in real time. The main task of face key point location is to perform key point calibration on the image of the face area located by the face detection model, and to judge the fatigue state by extracting the driver's fatigue characteristic parameters.

#### Feature extraction

**Figure 8:**
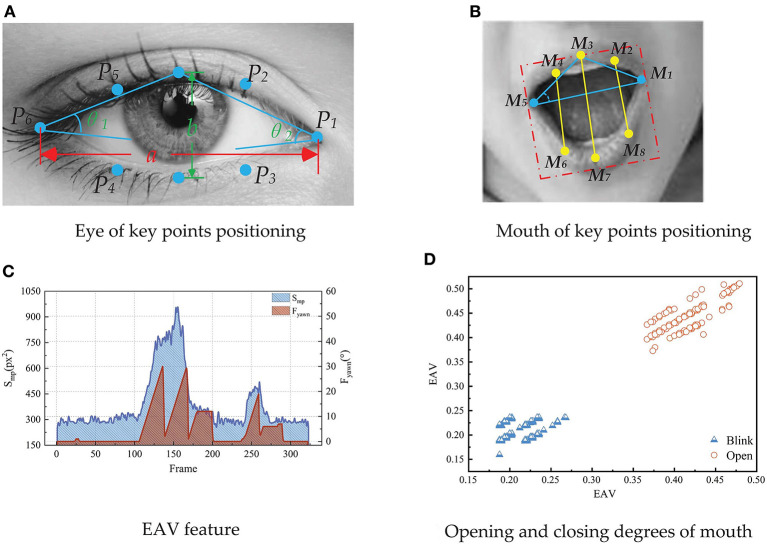
The feature of eye and mouth, including eye of key points positioning **(A)**, mouth of key points positioning **(B)**, EAV feature **(C)**, and opening and closing degrees of mouth **(D)**.

Based on the location of key points on the face of Dlib, the fatigue characterization parameters of the eyes and mouth are extracted. Whether the driver is in a fatigued driving state is characterized by extracting the blink frequency, EOA (eye opening angle), EAV (eye aspect vector), and the eye closure area parameters. The eye key point location is shown in Figure 8A.

The blink frequency refers to the ratio of the total number of frames of blinking images to the total number of images within a specified time window. Blinking is the movement of the eyes from opening to closing to opening. The blinking frequency can represent the driver's fatigue state. During normal driving, the driver blinks relatively fast, and the time consumed by each blink is about 0.2–0.3 s. In the fatigue driving state, the driver's eyes are dull, the blink time becomes longer, the duration is more than 1 s, and the blink frequency increases. Therefore, the blink frequency can intuitively represent the driver's fatigue state. The formula for calculating the blink frequency is:


$${\text{F}}_{\text{blink}}=\frac{\sum_{\text{i}}^{\text{N}}{\text{f}}_{\text{i}}}{\text{N}}$$

$\sum_{\text{i}}^{\text{N}}{\text{f}}_{\text{i}}$ is the number of images of the driver's eyes closed in a unit time, and **N** is the total number of images in a unit time.

The driver's eye opening and closing degree can be calculated according to the eye key point coordinates shown in Figure 8A. In this paper, EOA is used to represent the eye opening and closing degree parameter. The formula for calculating is as follows:


$$\text{EOA}=\text{arcsin}\left(\frac{\text{dis}\left(\text{P},{\text{P}}_{6}{\text{P}}_{1}\right)}{\text{dis}\left(\text{P},{\text{P}}_{6}\right)}\right)$$

**P**_**i**_**, i=1, 2, ⋯, 6** is the eye key point coordinate, and **dis** is the Euclidean distance between two eye key points.

The EAV is roughly constant when the eyes are open (23). When the driver blinks or closes the eyes, the EAV decreases rapidly. When the driver completes the blinking action, the EAV increases rapidly. The formula for calculating EAV is as follows:


$$\text{EAV}=\left(\frac{\left\|{\text{p}}_{5}-{\text{p}}_{4}\right\|}{\left\|{\text{p}}_{6}-{\text{p}}_{1}\right\|},\frac{\left\|{\text{p}}_{2}-{\text{p}}_{3}\right\|}{\left\|{\text{p}}_{6}-{\text{p}}_{1}\right\|}\right)$$

In order to simplify the calculation, the geometric shape fitted by the human eye feature points is approximately regarded as an ellipse in this paper. The driver's eye state can be characterized by the ratio of the ellipse area fitted by the key points of the eyes at a certain moment to the maximum value of the ellipse area fitted by the key points when the eyes are fully opened. The eye closed area ratio can be calculated as follows:


$${\text{S}}_{\text{ar}}=\frac{{\text{S}}^{\prime }}{{\text{S}}_{\text{max}}}=\frac{{\text{a}}^{\prime }{\text{b}}^{\prime }\pi }{{\text{a}}_{\text{max}}{\text{b}}_{\text{max}}\pi }$$

**S**^**′**^ is the fitting area of the eye at a certain moment, **S**_**max**_ is the maximum fitting area of the eye, **a**_**max**_ is the maximum canthus distance, **a**^**′**^ is the eyelid distance at a certain moment, **b**_**max**_ is the maximum eyelid distance, and **b**^**′**^ is the eyelid distance at a certain moment.

Figure 8C is drawn according to the driver's blink. It can be seen from the figure that in the state of eye opening and blinking, the vertical and horizontal dimensions of the eye have obvious cluster centers, which can distinguish the state of opening and blinking. Therefore, EAV can effectively distinguish the driver's eye opening and blinking state.

In addition to characterization parameters based on eye fatigue, yawning is also an important indicator for evaluating driver fatigue. Yawning is a deep breathing activity, a conditioned reflex in a state of fatigue. According to statistics, the duration of a yawn is about 4 s. When the driver speaks, the degree of mouth opening is constantly changing, but the duration is short. When the driver is fatigued, he will yawn with the characteristic of yawning. The mouth opening is obvious, and the duration is longer. In the process of yawning, the corners of the mouth will also have a significant change in opening and closing. Therefore, the extraction of mouth feature parameters is an effective supplement for judging fatigued driving.

There are obvious changes in the degree of mouth opening when the driver and the passenger speak, talk and other behaviors, which greatly interferes with the judgment of fatigue level based on the characteristics of the mouth. In order to distinguish the three state features of the driver yawning, speaking and mouth closed, the mouth state can be characterized by MAV (Mouth Aspect Vector), mouth corner opening and closing degree, and mouth opening area.

The frequency of yawning is defined as the number of image frames in which the driver yawns in unit time, and its calculation formula is as follows:


$${\text{F}}_{\text{yawn}}=\frac{\sum_{\text{k}}^{\text{N}}{\text{f}}_{\text{k}}}{\text{N}}$$

$\sum_{\text{k}}^{\text{N}}{\text{f}}_{\text{k}}$ is the number of frames of yawning within a certain period of time.

The calculation formula of MAV is as follows:


$$\text{MAV}=\left(\frac{\left\|{\text{M}}_{4}-{\text{M}}_{6}\right\|}{\left\|{\text{M}}_{5}-{\text{M}}_{1}\right\|},\frac{\left\|{\text{M}}_{3}-{\text{M}}_{7}\right\|}{\left\|{\text{M}}_{5}-{\text{M}}_{1}\right\|},\frac{\left\|{\text{M}}_{2}-{\text{M}}_{8}\right\|}{\left\|{\text{M}}_{5}-{\text{M}}_{1}\right\|}\right)$$

**M**_**i**_**, i=1, 2, ⋯, 6** is the eye key point coordinate. In the formula, the numerator is the distance vector between the key points of the upper lip and the lower lip, and the denominator is the distance vector of the key points of the corner of the mouth.

According to the coordinates of the key points of the mouth shown in Figure 8B, the mouth opening and closing degree of the driver can be calculated. In this paper, MOA (Mouth Opening Angle) is used to represent the mouth opening and closing degree parameters. It can be calculated as follows:


$${\text{S}}_{\text{mp}}=\left|{\text{x}}_{{\text{M}}_{8}}-{\text{x}}_{{\text{M}}_{1}}\right|•\left|{\text{y}}_{{\text{M}}_{7}}-{\text{y}}_{{\text{M}}_{3}}\right|$$

**x**_**M**_**8**__**, x**_**M**_**1**__ is the abscissa of the mouth key point of the corresponding serial number in Figure 8B, and **y**_**M**_**7**__**, y**_**M**_**3**__ is its ordinate.

The features including the driver's mouth closing, speaking or talking, and yawning are selected for analysis. The changes of mouth feature parameters are shown in Figure 8D. It can be seen from Figure 8D that there is a yawning process and a language conversation process. When yawning, the driver's mouth will quickly open to a certain angle and last for a long time. MOA will show an obviously exaggerated peak over time. When the driver speaks or talks, the changes of MOA is obviously different from the fluctuations of the characteristic parameters with the closed mouth or speaking.

Head pose estimation can more intuitively reflect the fatigue driving state (24). When the driver is in a normal driving state, there will be a slight change in the head posture. When in a state of fatigue, the driver's brain response ability is reduced. In addition to the characteristics of long blink time, the control and support ability of the head is reduced, and the head is often accompanied by nodding, tilting and other characteristics. When the driver frequently nods or tilts his head, it indicates that the fatigue state has reached a dangerous level, which is very likely to cause traffic accidents (25). Therefore, abnormal head posture is one of the important basis for judging the driver's fatigue driving.

Considering that the infrared camera is relatively fixed, the driver's head is abstracted into an intangible rigid object to facilitate the estimation of the characteristic state of the driver's head. The characteristic of the rigid body is that the relative position of its internal feature points does not change. When the rigid body is in motion, the shape and size of the object itself does not change, and the object itself only performs rotation and translation transformations.

**Figure 9:**
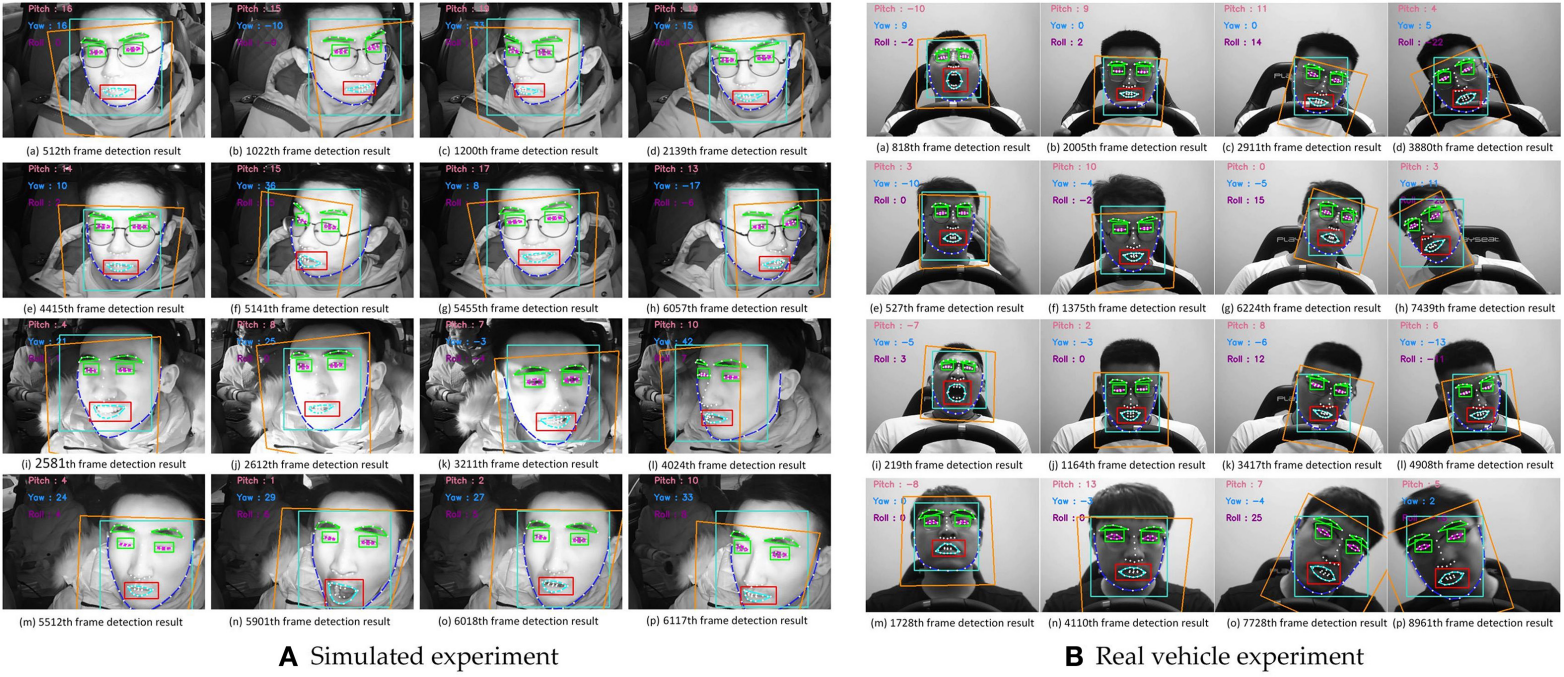
Head pose estimation results of simulated experiment **(A)** and real vehicle experiment **(B)**.

Head pose estimation can be performed using the EPnP (26) method, which is based on MTCNN to identify five 2D landmarks on the face. Through the rotation matrix of the camera, the three-dimensional point distribution in the world coordinate system can be mapped to the two-dimensional image feature point distribution, and the rotation matrix can be converted into the Euler angle feature parameters of the head pose. EPnP is a high precision and fast pose estimation method with strong anti-interference ability. The effects of extracting the driver's head feature parameters in the simulated driving environment and the real vehicle environment are shown in Figure 9.

The light blue rectangle represents the driver's face area, and the orange rectangle represents the driver's face orientation. The Euler angle solution results are displayed at the upper left of the image for easy information acquisition. The results show that the algorithm can estimate the heads of different postures in the real vehicle-driving environment and the simulated driving environment, and has a good anti-interference ability against day, night and abnormal lighting.

## Results and discussions

### Data

The study found that most drivers' fatigue index data could be observed for 60 s or more to obtain reasonable signs of driver fatigue (27). Therefore, the experts screened 1-min time segments with typical fatigue characterization phenomena in the segmented data, and constructed a fatigue driving dataset. If there is no obvious fatigue feature in the frequency data after 5 min segmentation, the 1 min data is selected as the normal driving data set. In order to improve the quality and reliability of the fatigue label data, the experts revised the scores through the playback of the video process and confirmed their final scores. Due to the physical strength of the subjects and the irregular driving posture, the experimental data of nine drivers were excluded. Thirty-eight groups of valid video data were selected to construct a fatigue-driving database, including 23 groups of normal driving experimental data and 15 groups of fatigue driving experimental data.

Eleven features such as eyes, mouth and head in the driver's driving behavior are considered as candidate feature parameters.

During data collection, due to the irregular driving behavior of experimental equipment or experimenters, noise, errors and other data are introduced in the data collection process. These data will have a great impact on the model during the model training process, and some even directly affect the effect of the model. Data cleaning refers to straightening out the messy original data. It is the process of changing ‘dirty data' into ‘clean data' and correcting errors in the original data. It is the cornerstone of the entire data analysis process. In order to avoid “dirty data” being directly input into the model for training and verification, which affects the recognition effect of the model, it is necessary to perform data cleaning operations on these data. There are many good methods to do this well (28–31). The Fast Fourier Transform (FFT) algorithm is used to denoise the data (32).


$$\text{X}\left(\text{k}\right)=\sum_{\text{n}=0}^{\text{N}-1}\text{a}\left(\text{n}\right){\text{e}}^{-j\frac{2\pi \text{k}}{\text{N}}\text{n}},0\leq k\leq N-1$$

**a**(**n**) is a discrete finite-length sequence of length *M*, $\text{M},{\text{e}}^{-\text{j}\frac{2\pi k}{\text{N}}\text{n}}$ is the twiddle factor, **X**(**k**) is the N-point DFT of **a**(**n**), and **N** is the transform length, **N**≥**M**.

Divide **a**(**n**) into two groups according to the parity of the serial number n, and rewrite the Fourier transform of equation as:


$$\begin{array}{l} \text{X}\left(\text{k}\right)=\sum_{n=0}^{\frac{\text{N}}{2}-1}\text{a}(2\text{n}){\text{e}}^{-\text{j}\frac{2\pi \text{k}}{\text{N}}2\text{n}}+\sum_{\text{n}=0}^{\frac{\text{N}}{2}-1}\text{a}(2\text{n}+1){\text{e}}^{-\text{j}\frac{2\pi \text{k}}{\text{N}}(2\text{n}+1)}\\ \;\text{n}=0,1,\cdots ,\frac{\text{N}}{2}-1 \end{array}$$

**Figure 10:**
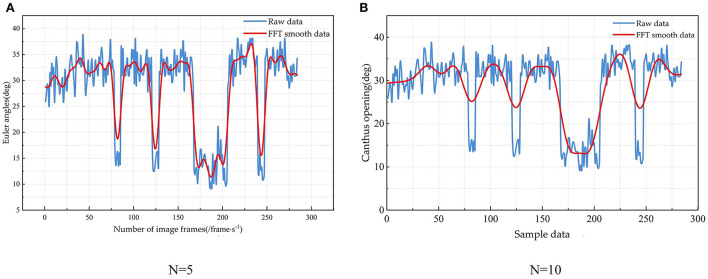
Filtering results—FFT with filter windows of 5 **(A)** and 10 **(B)**.

In specific applications, the size of the filtering window affects the final effect of data smoothing. If the window is too large, the curve is flat, and the filtered data differs greatly from the original data, which may lead to unrealistic data. If the window is too small, there may be overfitting. Combined with the amount of self-built fatigue driving data, two filter window lengths **N=10** and **N=5** are selected for comparison, as shown in Figure 10.

The curve is smooth in Figure 10B, but the peak value is not obvious enough. The difference with the original data is too large. It is not easy to extract features. The filtering curve in Figure 10A is ideal, and it is easy to select feature points from it. The amount of experimental data is small, and the filter data changes significantly when **N=5** in this paper. This makes it possible to express the fluctuation of the original data well, and the difference with the original data is small. Therefore, an FFT filter with a filter window length of 5 is chosen in this paper.

The experimental data is preprocessed according to the above method, and 18835 groups of simulated driving experimental data are obtained, of which 16612 groups are valid data. According to the time series, 9113 sets of data are selected from the driving data to form the original database for model calibration and training. 3275 sets of data are used for model testing, and the remaining 4224 sets of data are used for model validation. Factor analysis is used to extract the main factors of the reaction fatigue identification model to the greatest extent. A factor analysis model is constructed based on the analysis of the preprocessed experimental data to achieve the purpose of dimensionality reduction and avoid the dimensional “disaster.”

The percentage of squared and variance of the loading and the information content of each factor are shown in Tables 1, 2. The analysis results show that the cumulative variance contribution rate of the first six factors is 95.867%, which is more than 90%. It represents most of the information of all parameters. Considering the information content of each factor and the bending point of the gravel diagram, the first six common factors are selected as the main features of fatigue driving state identification.

**Table 1:** Total explained variance.

**Component**	**Eigenvalues**	**Percent of extracted**	**Cumulative**
		**loading squared and**	**amount of**
		**variance (%)**	**information (%)**
1	3.698	33.617	33.617
2	2.598	23.615	57.232
3	1.466	13.331	70.563
4	1.251	11.376	81.939
5	0.903	8.212	90.152
6	0.629	5.716	95.867
7	0.271	2.462	98.329
8	0.098	0.890	99.219
9	0.051	0.468	99.687
10	0.023	0.207	99.894
11	0.012	0.106	100.000

**Table 2:** Loadings matrix of rotated factor.

**Parameter**	**Component**										
	**1**	**2**	**3**	**4**	**5**	**6**	**7**	**8**	**9**	**10**	**11**
EAV	0.968	0.031	– 0.200	– 0.054	0.018	– 0.015	– 0.002	0.023	– 0.002	– 0.089	– 0.104
MAV	0.080	0.955	– 0.053	– 0.122	0.017	– 0.011	– 0.060	0.090	– 0.226	0.002	0.000
*S*_*ar*_	0.961	0.038	– 0.196	– 0.126	0.036	– 0.025	0.023	0.017	0.000	– 0.081	0.110
*S*_*mp*_	0.007	0.614	– 0.089	– 0.204	– 0.016	– 0.031	0.003	0.756	0.001	– 0.002	0.000
Pitch	0.091	– 0.135	0.027	0.237	0.109	– 0.014	0.951	0.001	– 0.002	0.003	0.000
Yaw	– 0.022	– 0.080	0.009	0.961	0.000	0.062	0.233	– 0.105	0.006	0.006	– 0.001
Roll	0.065	0.087	0.007	0.001	0.988	0.049	0.097	– 0.007	0.003	0.001	0.000
EOA	0.936	0.042	– 0.188	0.171	0.051	– 0.003	0.113	– 0.031	– 0.004	0.201	– 0.003
MOA	0.017	0.949	– 0.053	0.055	0.108	0.022	– 0.111	0.125	0.232	– 0.001	0.000
*F*_*blink*_	– 0.244	– 0.059	0.964	0.007	0.004	0.017	0.015	– 0.031	– 0.001	– 0.002	– 0.001
*F*_*yawn*_	– 0.027	0.002	0.025	0.055	0.048	0.996	– 0.012	−0.015	0.001	0.000	0.000

### Model

**Figure 11:**
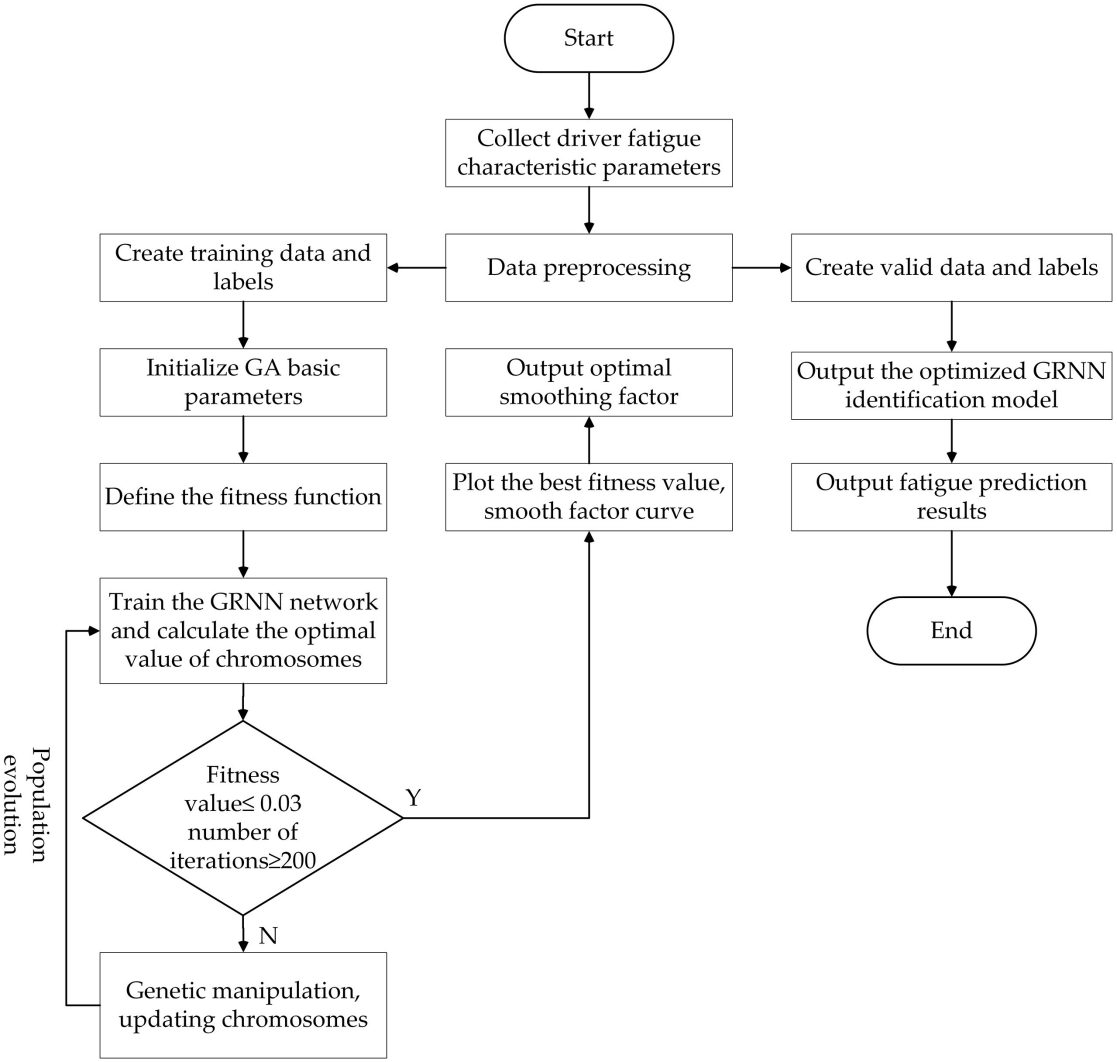
Training flowchart of GA optimizes GRNN network.

In order to improve the learning efficiency and identification accuracy of the GRNN identification model, a generalized neural network model optimized by genetic algorithm GA-GRNN neural network model is proposed in this paper. The optimal fitness value and the hidden layer smooth factor **σ** of the generalized regression neural network are found in the search space through GA, which avoids the influence of manual adjustment of the smooth factor and improves the performance of the neural network. The GA-GRNN model training process is shown in Figure 11.

Generalized Regression Neural Network is a RBF Radial Basis Neural Network proposed by Professor D.F. Specht. The output function of the network is **y**^**A**^, and the calculation formula is as follows:


$${\text{y}}^{\text{A}}=\frac{\sum_{\text{i}=1}^{\text{n}}{\text{Y}}_{\text{i}}^{\text{A}}\text{ exp}\left[-\frac{{\left(\text{X}-{\text{X}}_{\text{i}}^{\text{A}}\right)}^{\text{T}}\left(\text{X}-{\text{X}}_{\text{i}}^{\text{A}}\right)}{2\sigma^{2}}\right]}{\sum_{\text{i}=1}^{\text{n}}\text{exp}\left[-\frac{{\left(\text{X}-{\text{X}}_{\text{i}}^{\text{A}}\right)}^{\text{T}}\left(\text{X}-{\text{X}}_{\text{i}}^{\text{A}}\right)}{2\sigma^{2}}\right]}$$

${\text{X}}_{\text{i}}^{\text{A}}$ is the i-th data in data set A, ${\text{Y}}_{\text{i}}^{\text{A}}$ is the label corresponding to the i-th data in dataset A. **y**^**A**^ is the output function of the network, which is the estimated value of ${\text{Y}}_{\text{i}}^{\text{A}}$ by the weighted average operation.

**Figure 12:**
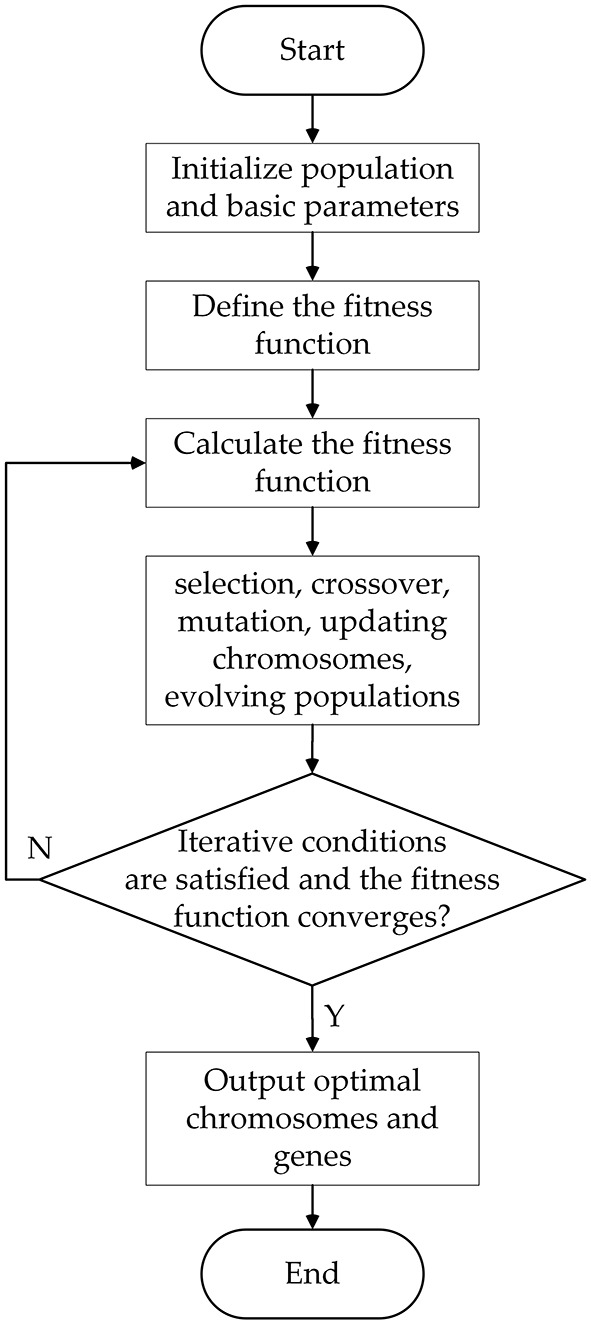
Optimization process of genetic algorithm.

The genetic algorithm is selected to optimize the generalized regression neural network model to improve the identification accuracy and generalization ability of the model. The evolution process of the genetic algorithm population mainly includes initializing the population, selection, crossover and mutation. The optimization process is shown in Figure 12.

The specific optimization process is as follows:

Step 1: Initialize basic parameters. The number of chromosomes in the population is 80, the crossover probability is 0.7, the mutation probability is 0.25, and the maximum number of evolutionary times is 200, σ ∈ [**0, 2]**.
Step 2: Define the fitness function. The mean square error between the output value of the GA-GRNN neural network and the actual value of the data is defined as the population fitness function. The smooth factor **σ** is mapped to the fitness function, and the GRNN fatigue driving state identification model is constructed. The calculation formula of the fitness function is as follows:
  $$\text{MSE}\left(\sigma \right)=\frac{1}{\text{n}}\sum_{\text{i}=1}^{\text{n}}{\left({\text{y}}^{\text{A}}\left(\sigma ,{\text{X}}_{\text{i}}^{\text{A}}\right)-{\text{Y}}_{\text{i}}^{\text{A}}\right)}^{2}$$
Step 3: Train the GRNN network. Since the fatigue driving state is based on the continuity of the fatigue representation phenomenon in the time series, it is unreasonable to determine the fatigue driving only from the recognition results of a single data or image frame. Therefore, t the sliding window algorithm is used to train the model. The training samples and label data are input into the GRNN to train the model. The length of the sliding window is a key parameter that affects the accuracy of the identification model. If the window length is too short, the obtained time series may not fully cover the fatigue state. If the time window is too long, the sequence will contain too much redundant information, which will reduce the recognition accuracy of the model. Considering the accuracy and calculation speed of the balanced identification model, after many experiments, the final window size is 900 and the sliding step is 80.
Step 4: Determine whether the fitness value and iteration conditions are satisfied. It is reasonable to take the fitness threshold (0.01, 0.0533) according to the references (33) and (34). Therefore, set the fitness value of 0.03 as the judgment condition. If the average fitness value of the population is < 0.03 and the number of iterations of the population reaches 200, the GA GRNN fatigue driving identification model can be output. Otherwise, the population is updated and iterated until the judgment condition is met.
Step 5: Population evolution. Update the population, update chromosomes or genes through evolutionary methods of selection, crossover, and mutation, and select excellent individuals that are superior to the previous generation until the optimal individual is selected.
Step 6: GA optimization ends. Output the optimized GRNN model, save the trained GA-GRNN identification model, and output the best smooth factor.

### Model training, test, validation and evaluation

The 12,423 sets of driving data processed by FFT are used for model calibration and training, and the remaining 4,189 sets of data are used for model validation. The optimal smooth factor can be found through the genetic algorithm to obtain the best fitness value. When the GA iteration reaches 125, the population is no longer updated. At this time, the average fitness value is 0.2385. When the iteration reaches 136 times, the optimal fitness value is reached, which is 0.0429. When the GA-GRNN identification model reaches the iterative condition, the optimal smoothing factor is 0.134. The identification accuracy of the GRNN model optimized by the genetic algorithm is 95.7%, and the identification accuracy of the original GRNN model after manual parameter adjustment is 88.9%, which is 6.8% higher than that of the GRNN identification model before optimization.

It compares the GRNN identification model before and after optimization on the training data set through 10 rounds of K-fold cross validation, where the original GRNN model uses manual intervention to adjust the smoothing factor.

In order to test the accuracy of the GRNN identification model optimized by the GA algorithm, 3275 sets of test data and labels are used to test the GA-GRNN model, and 10 rounds of 5-fold cross-validation are used to test the GA-GRNN and the manually adjusted GRNN model. The results show that the accuracy rate of GRNN identification model is 88.7%, and the average identification accuracy of GA-GRNN model is only 94.1% after 10 rounds of 5-fold cross-validation test. The reason is that most of the states in the test set can be correctly detected, but the accuracy of the identification of abnormal head posture data is low. This is because in the test set, there are some data under the characteristics of bowing, tilting, etc. Also labeled as fatigue state data, this situation is difficult to identify relative to the GA-GRNN identification model.

Considering the objective and multi-dimensional measurement model indicators, this paper uses simulated driving data and real vehicle driving data to verify the trained GA-GRNN model. In order to verify the superiority of the optimized recognition algorithm, at the same time, the random forest, K-nearest neighbor algorithm and the GA-GRNN model are introduced for comparison. Random Forest is a representative bagging method, which is a kind of ensemble algorithm. The base evaluator of random forest is a decision tree model. The core idea of the bagging method is to construct multiple independent base evaluators, and decide the classification result of the final integrated evaluator through the principle of voting or majority voting. This model reduces the problem of easy overfitting of decision tree models to a certain extent. At the same time, the principle is simple and the operation is easy, so it is widely used in data analysis, processing, and other fields. K-nearest neighbors is a supervised learning model. The data can be predicted with majority voting, and it can be judged that it belongs to K categories closest to the data. The two most important factors of the KNN algorithm are the choice of the K value and the calculation of the distance. KNN has the advantages of simple principle and good classification effect, and is widely used in feature classification tasks.

The four evaluation indicators of accuracy, recall, precision and F1-score were used to evaluate GA-GRNN, GRNN, RF and KNN. The performance of the nearest neighbor model. Accuracy is the percentage of correctly identifying fatigue and non-fatigue driving behaviors in the total number of experimental data behaviors or the probability of correct identification. It can be calculated as follows:


$$\text{Accuracy}=\frac{\text{TP}+\text{TN}}{\text{FP}+\text{FN}+\text{TP}+\text{TN}}\times 100\%$$

TP is the number of true positive samples. TN is the number of true negative samples. FP is the number of false positive samples. FN is the number of false negative samples.

Recall is the ratio of the number of correctly classified positive samples to the total number of positive samples. There is a trade-off between recall and precision, and the balance between the two represents a balance between the need to capture the minority class and the need to avoid misjudging the majority class. It can be calculated as follows:


$$\text{Recall}=\frac{\text{TP}}{\text{TP}+\text{FN}}\times 100\%$$

Precision is the ratio of the number of correctly classified positive samples to the total samples predicted by the model to driver normally. Precision represents a measure of the cost of misjudging the majority class. It can be calculated as follows:


$$\text{Precision}=\frac{\text{TP}}{\text{TP}+\text{FP}}\times 100\%$$

F1-score is used to reconciling the balance between precision and recall. The value range of F1-score is [0, 1]. The higher the score, the better the performance. It can be calculated as follows:


$$\text{F}1-\text{score}=\frac{2\times \text{Pre}\times \text{Rec}}{\text{Pre}+\text{Rec}}\times 100\%$$

The remaining 4,224 sets of data and labels are used for the validation of the trained GA-GRNN model. The experimental results are shown in Table 3.

**Table 3:** Validation results of the model.

**Model predictions**	**Real data results**							
	**GRNN**		**KNN**		**RF**		**GA-GRNN**	
	**Normal**	**Fatigue**	**Normal**	**Fatigue**	**Normal**	**Fatigue**	**Normal**	**Fatigue**
	**driving**	**driving**	**driving**	**driving**	**driving**	**driving**	**driving**	**driving**
Normal driving	1629	241	1705	254	1797	193	1671	157
Fatigue driving	214	2117	228	2037	154	2080	127	2269

The verification results of the four types of identification model experiments are organized, and the comparison results are shown in Supplementary Figure 5. The verification results show that the GRNN model has the lowest recognition accuracy of fatigue driving and the worst generalization ability. The accuracy of KNN model is 90.4%, the recall is 87.0%, the precision is 88.2%, and the F1-score is 87.6%, which is not as good as the performance of GA-GRNN model. The accuracy of RF model is 93.0%, the precision is 92.1%, the F1-score is 91.2%, but the recall is only 90.3%, and the overall performance of the model is unstable. The accuracy of GA-GRNN model is 93.3%, the recall is 91.4%, the precision is 92.9%, and the F1-score is 92.1%. Compared with GRNN, KNN, and RF identification models, the algorithm proposed in this study improves the accuracy of fatigue driving state identification. Compared with before optimization, the recognition accuracy of GA-GRNN model is increased by 4.7%, the recall rate is increased by 4.7%, the precision rate is increased by 5.2%, and the F1-score value is increased by 4.9%. From the confusion matrix of the identification results in Table 3, it can be seen that in the identification results of the four models, the number of results misjudging normal driving as fatigue driving is more than that of fatigue driving being misjudged as normal driving, which may be related to our assumption. It is related to the specified fatigue detection threshold. We divided the experimental data set with the expert scoring method. When performing manual annotation, the driver's relatively awake state is also identified as fatigue driving. Nevertheless, the driver's fatigue characteristics are usually not particularly obvious in this state. A driver with even the slightest sign or characteristic of fatigue is judged as fatigue driving. In the identification results of KNN and GRNN models, the misjudgements of both normal driving and fatigue driving are more than the RF and GA-GRNN models, and the fatigue detection performance in this study is poor. Although the RF model can accurately predict the state of normal driving, it has certain defects. For example, many fatigue driving are misjudged as normal driving, and many fatigue driving results are misjudged by normal driving. The number of correctly predicted fatigue driving results is less than that of GA-GRNN model, so the overall performance of RF model is not as good as GA-GRNN model. The number of fatigue results correctly identified by GA-GRNN model is the largest, and the number of identification results of the fatigue driving and the normal driving also shows the superiority of GA-GRNN model in fatigue identification.

## Conclusions

Fatigue driving poses a serious threat to the safety of pedestrians and drivers. Fatigue driving identification has become a key research topic at home and abroad. A fatigue driving state identification method based on GA-GRNN is proposed in this paper. Firstly, the trend changes of the traffic environment and travel safety and the hazards caused by fatigue driving are expounded, which leads to the urgent need and importance of developing a real-time monitoring, high precision, non-invasive fatigue identification model. Secondly, the definition of fatigue driving and the characterization phenomenon when fatigue occurs are introduced. Thirdly, focusing on the construction method of the fatigue driving state identification model, this paper introduces the research status and achievements of domestic and foreign scholars in this field. Through the review of previous studies, the advantages and disadvantages of different fatigue identification methods are analyzed. Finally, the research ideas, methods and technical routes of this system are proposed. The main work of this paper includes the following aspects:

1) Design and organize simulated driving experiments and real vehicle driving experiments. 33 participants are recruited to collect the experimental data of normal driving and fatigue driving. After the experiment, the experts determined the driver's fatigue level according to the KSS scale and recorded the driver's label data in different states. Based on this, a fatigue driving sample database is established, which is used for the analysis of fatigue characterization parameters, the construction and calibration of fatigue identification model.
2) The architecture of the improved MTCNN face detection model is introduced, and the performance advantages of the MTCNN model before and after the improvement are compared. The improved MTCNN network is applied to face detection and localizes the driver's face region. The Dlib library is used to extract the coordinate values of 68 key points of the face, which are used to collect the characteristic parameters of the driver's eyes and mouth. These parameters include blink frequency, eye angle opening and closing, eye vertical and horizontal amount, eye closed area, yawn frequency, mouth opening and closing degree, mouth vertical and horizontal amount, and mouth open area. According to the correspondence between the coordinates of the 2D facial key points and the 3D face model coordinate system, the Euler angle parameters of the head pose are calculated. Abnormal head postures are divided into nodding and tilting, which were used as fatigue characterization parameters in this paper. By converting image information into data information, the data fluctuation changes of each characteristic parameter when fatigue driving behavior occurs are analyzed. The problem of poor stability and robustness of a single parameter identification model due to individual differences is compensated by the extraction of multi-feature parameters.
3) The raw experimental data are pre-processed using the Fast Fourier algorithm. A factor analysis model is constructed from the processed data. The main factors satisfying the conditions are extracted as the main features when the model is built to reduce the data dimension. Generalized regression neural network has the advantages of strong nonlinear mapping and strong fault tolerance. The genetic optimization algorithm can help the generalized regression neural network to converge quickly, improve the learning speed of the model, and find the optimal smooth factor. A GA-GRNN fatigue driving identification model is constructed combining the two algorithms. Compared with KNN, RF, and GRNN fatigue driving identification models, the performance and generalization ability of the model are verified in multiple dimensions on the verification data set. The verification results show that the GA GRNN identification model has the best generalization ability and high stability. This research demonstrates the feasibility of machine vision technology for fatigue driving detection, and provides new methods and technical support for real-time and accurate identification of fatigue driving state.

## Data availability statement

The original contributions presented in the study are included in the article/Supplementary material, further inquiries can be directed to the corresponding author.

## Ethics statement

The studies involving human participants were reviewed and approved by Ethics Committee of the college of Electromechanical Engineering, Qingdao University of Science and Technology. The patients/participants provided their written informed consent to participate in this study. Written informed consent was obtained from the individual(s) for the publication of any potentially identifiable images or data included in this article.

## Author contributions

Conceptualization, methodology, validation, and resources: XW and LC. Software: LC and YZ. Formal analysis: LC, JH, and GW. Investigation: LC and HS. Data curation: LC, YZ, and HS. Writing—original draft preparation: LC. Writing—review and editing: XW, LC, and JH. Visualization: GW, FZ, and QW. Supervision, project administration, and funding acquisition: XW. All authors contributed to the article and approved the submitted version.

## Funding

This research was funded by the Natural Science Foundation of Shandong Province (ZR2020MF082), the Collaborative Innovation Center for Intelligent Green Manufacturing Technology and Equipment of Shandong Province (IGSD-2020-012), the Qingdao Top Talent Program of Entrepreneurship and Innovation (19-3-2-8-zhc), and the National Key Research and Development Program (2018YFB1601500).

## Conflict of interest

The authors declare that the research was conducted in the absence of any commercial or financial relationships that could be construed as a potential conflict of interest.

## Publisher's note

All claims expressed in this article are solely those of the authors and do not necessarily represent those of their affiliated organizations, or those of the publisher, the editors and the reviewers. Any product that may be evaluated in this article, or claim that may be made by its manufacturer, is not guaranteed or endorsed by the publisher.
