# Supplementary material for: A real-time driver fatigue identification method based on GA-GRNN
Source: Front Public Health. 2022 Oct 20;10:991350. doi: 10.3389/fpubh.2022.991350 (PMC9632354; doi:10.3389/fpubh.2022.991350)
Supplement: Supplementary file 1 [file Data_Sheet_1.ZIP › Supplementary_Material.pdf]

Supplementary Material

1 Supplementary Description of the Algorithm

1.1 The improved MTCNN face detection algorithm

The MTCNN face detection algorithm can be divided into three subtasks, which are face or non-face classification, face candidate box regression, and face feature point localization.

Face classification is the first subtask of MTCNN. The cross-entropy loss function is used as the evaluation metric, which can be calculated by the following formula:

$$L_i^{det} = -(y_i^{det} \log(p_i) + (1 - y_i^{det})(1 - \log(p_i)))$$

L_i^{det} is the probability that the i-th image of the input is a face. p_i is the probability that the sample x_i is predicted to output a face through the network. y_i^{det} is the ground-truth label of the image sample, $y_i^{det} \in \{0, 1\}$.

IoU is a numerical value that measures the accuracy of the face detection model. The diagram of IoU is shown in Figure 1. $Face\_t$ is the area of the face candidate frame and $Face\_p$ represents the area of the face frame predicted by the model. The area where the two areas intersect is the IoU, which can be calculated as the following formula:

$$IoU = \frac{A(Face\_t \cap Face\_p)}{A(Face\_t \cup Face\_p)}$$

$A(Face\_t \cap Face\_p)$ is the area where $Face\_t$ and $Face\_p$ intersect. $A(Face\_t \cup Face\_p)$ is the area where $Face\_t$ is merged with $Face\_p$.

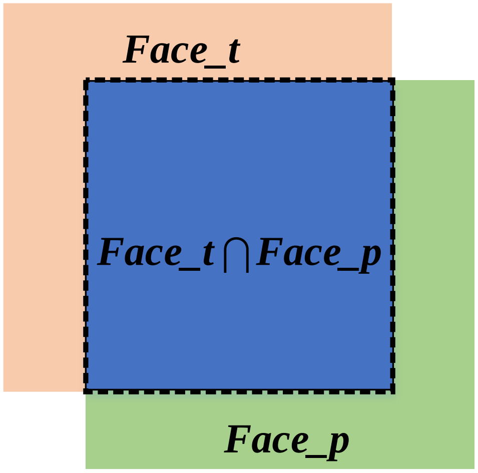

Figure 1. Diagram of intersection over union

In the face detection box regression task, the offset between the candidate box and the ground-truth box is used for prediction. The improved bounding box loss function is used in this paper, and the $\boldsymbol{IoU}$ value is added to the loss function. The calculation formula is as follows:

$$L_i^{box} = 1 - IoU + \frac{\rho^2\left(b^{pb}, b^{gt}\right)}{c^2} + \frac{\frac{4}{\pi^2}\left(arctan\frac{w^{gt}}{h^{gt}} - arctan\frac{w^{pb}}{h^{pb}}\right)^4}{(1 - IoU) + \frac{4}{\pi^2}\left(arctan\frac{w^{gt}}{h^{gt}} - arctan\frac{w^{pb}}{h^{pb}}\right)^2}$$

When $IoU = 1$, the predicted face frame overlaps with the real face frame. $\rho^2\left(b^{pb}, b^{gt}\right)$ is the square of the Euclidean distance between the predicted face frame and the real face frame. c is the diagonal length of the minimum bounding box formed by the predicted face frame b^{pb} and the real face frame b^{gt}. w^{gt} and h^{gt} are the width and height of the real face frame. w^{pb} and h^{pb} are the width and height of the predicted face frame.

The function of face feature points is to correct the face, so that the face area can be effectively detected in the case of tilting the head, side face, etc. The calculation formula of the loss function of the feature point is as follows:

$$L_i^{landmark} = \left\|\hat{y}_i^{landmark} - y_i^{landmark}\right\|_2^2$$

$\hat{y}_i^{landmark}$ is the face feature coordinate point predicted by the network for the i-th sample, which is a ten-dimensional vector. $y_i^{landmark}$ is the actual face landmark of the i-th image sample.

Since the input image is composed of various types of images, such as small-sized faces, faces in different scene environments, and aligned face images. During training, the subtask part of the loss function is no longer used for different types of input images in the model. For example, the loss function L_i^{det} is used as the background learning process, and the other two loss functions are set to 0. The calculation process can be simplified in this way. The loss function for the entire model training can be calculated as follows:

$$min\sum_{i=1}^{n}\sum\nolimits_{j\in\{det,box,landmark\}}\alpha_j\beta_i^jL_i^j$$

N is the number of training tasks. α_j is the weight of the j-th sub-network in the overall loss. L_i^j represents three different loss functions.

α_{det}, α_{box} and $\alpha_{landmark}$ are set to 1, 0.5, and 0.5 in the P-Net and R-Net, and they are set to 1, 0.5, and 1 in O-Net. β_i^j is the sample label, and the value of it is {0,1}. As can be seen from the weight size distribution of α_j, the training tasks focus on face classification and face bounding box regression in the P-Net and R-Net. In the O-Net, the training task focuses on the coordinates of facial feature points.

1.2 The face key point localization algorithm based on Dlib

After locating the driver's face area, the driver's facial feature parameters will be extracted in the face area. In order to ensure that the recognition model can run smoothly in embedded devices with small size and relatively weak performance, the face alignment model interface packaged in the Dlib library is used to locate the key points of the driver's face, which makes it possible to judge the driver's fatigue driving state in real time. The algorithm is less time-consuming and efficient, and meets the needs of real-time positioning of face key points.

Compared with other face alignment models, the advantage of Dlib algorithm is that it is an open source SDK toolkit developed based on C++ language. It is also a general learning framework that is not limited by platforms, which is easy to transplant to embedded systems and has strong compatibility. In addition, the Dlib algorithm is also used in different research directions such as machine learning, cloud solutions, Internet of Things and embedded systems, and has been used in both academic and industrial fields. Among them, 68 key points are the most widely used, and the serial number of face key points is shown in Figure 2.

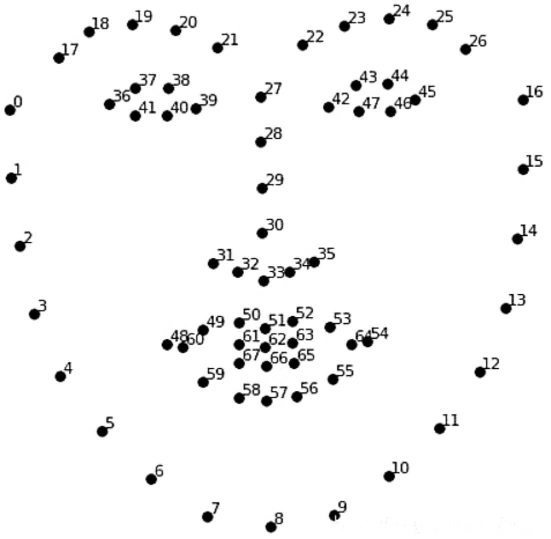

Figure 2. Number of 68 face key point

ERT (Ensemble of Regression Trees) is used in Dlib library to locate the key points of the face. ERT is a key points detection model based on regression method. The algorithm continuously reduces the residual between the predicted face shape and the real face shape by building a cascaded GBDT (Gradient Boosting Decision Tree), so that the position of the face feature points gradually returns to the real position. When the established model achieves the optimal detection accuracy, the purpose of face alignment is achieved. The basic principles of face alignment model based on ensemble regression tree are as follows:

$$\hat{\boldsymbol{S}}^{(t+1)} = \hat{\boldsymbol{S}}^{(t)} + \boldsymbol{r_t}(\boldsymbol{V}, \hat{\boldsymbol{S}}^{(t)})$$

$\boldsymbol{S_k^{(t)}}$ is the face shape estimation result predicted by the current iteration. $\boldsymbol{S_k^{(t+1)}}$ is the estimated face shape for the t-th iteration. $\boldsymbol{r_t}(\boldsymbol{V}, \hat{\boldsymbol{S}}^{(t)})$ is the residual regressor or coordinate calculated for the current layer. $\boldsymbol{V}$ is the input image. $\boldsymbol{r_t}$ represents the current cascade regressor. The residual value of the face shape vector can be calculated by using cascaded regressors. The calculation results of multiple weak

regressors can be integrated, and all feature points can be iteratively updated. This gradually reduces the error between the coordinate value of feature point predicted by the model and the coordinate value of the real face feature point, which can improve the positioning accuracy of the face feature point.

The specific process of ERT algorithm is as follows:

(1) Establish ERT model

The vector $\boldsymbol{S}$ can be used to describe the face shape vector:

$$\boldsymbol{S} = (\boldsymbol{x}_1^T, \boldsymbol{x}_2^T, \cdots, \boldsymbol{x}_p^T)^T$$

$\boldsymbol{x_1}, \boldsymbol{x_2}, \cdots, \boldsymbol{x_p}$ is the two-dimensional coordinate of the p-th feature point in the image. $\boldsymbol{S}$ is the shape vector formed by the p-th face feature point.

Create a triplet array $\{\left(\boldsymbol{V}_{\boldsymbol{\pi_m}}, \widehat{\boldsymbol{S}}_{\boldsymbol{m}}^{(\boldsymbol{0})}, \Delta\boldsymbol{S}_{\boldsymbol{m}}^{(\boldsymbol{0})}\right)\}_{\boldsymbol{m=1}}^{\boldsymbol{N}}$ containing the training image of face landmarks, the initial face shape, and the target face shape. The formula for calculating the difference between the real face shape vector and the model estimated face shape vector is as follows:

$$\boldsymbol{\pi_m} \in \{\boldsymbol{1}, \boldsymbol{2}, \cdots, \boldsymbol{n}\}$$

$$\begin{cases} \widehat{\boldsymbol{S}}_{\boldsymbol{m}}^{(\boldsymbol{0})} \in \{\boldsymbol{S_1}, \boldsymbol{S_2}, \cdots, \boldsymbol{S_n}\} \backslash \boldsymbol{S}_{\boldsymbol{\pi_m}} \\ \Delta\boldsymbol{S}_{\boldsymbol{m}}^{(\boldsymbol{0})} = \boldsymbol{S}_{\boldsymbol{\pi_m}} - \boldsymbol{S}_{\boldsymbol{m}}^{(\boldsymbol{0})} \end{cases}$$

$\boldsymbol{n}$ is the number of images. $\Delta\boldsymbol{S}_{\boldsymbol{m}}^{(\boldsymbol{0})}$ represents the difference between the real shape vector of the image and the face shape vector estimated by the model during initialization. $\boldsymbol{S}_{\boldsymbol{\pi_m}}$ is the real face shape. $\boldsymbol{S}_{\boldsymbol{m}}^{(\boldsymbol{0})}$ represents the initialized face shape vector.

For other regressors $\boldsymbol{r_i}$ in the cascade, there are as follows:

$$\widehat{\boldsymbol{S}}_{\boldsymbol{m}}^{(\boldsymbol{t+1})} = \widehat{\boldsymbol{S}}_{\boldsymbol{m}}^{(\boldsymbol{t})} + \boldsymbol{r_i}(\boldsymbol{V}_{\boldsymbol{\pi_m}}, \widehat{\boldsymbol{S}}_{\boldsymbol{m}}^{(\boldsymbol{t})})$$

$$\Delta\boldsymbol{S}_{\boldsymbol{m}}^{(\boldsymbol{t+1})} = \boldsymbol{S}_{\boldsymbol{\pi_m}} - \widehat{\boldsymbol{S}}_{\boldsymbol{m}}^{(\boldsymbol{t+1})}$$

(2) Learn weak regressors

There is a dataset $\{\left(\boldsymbol{V}_{\boldsymbol{\pi_m}}, \widehat{\boldsymbol{S}}_{\boldsymbol{m}}^{(\boldsymbol{0})}, \Delta\boldsymbol{S}_{\boldsymbol{m}}^{(\boldsymbol{0})}\right)\}_{\boldsymbol{m=1}}^{\boldsymbol{N}}$ to be trained, and the learning rate is set to (0,1) . Initialize one of the tree models as follows:

$$\boldsymbol{f_0}(\boldsymbol{V}, \widehat{\boldsymbol{S}}^{(t)}) = \boldsymbol{argmin} \sum_{\boldsymbol{m=1}}^{\boldsymbol{N}} \left\| \Delta\boldsymbol{S}_{\boldsymbol{i}}^{(\boldsymbol{t})} - \boldsymbol{\gamma} \right\|^{\boldsymbol{2}}$$

$$\boldsymbol{r_{mk}} = \Delta\boldsymbol{S}_{\boldsymbol{m}}^{(\boldsymbol{t})} - \boldsymbol{f_{k-1}}(\boldsymbol{V}_{\boldsymbol{\pi_m}}, \widehat{\boldsymbol{S}}_{\boldsymbol{m}}^{(\boldsymbol{t})})$$

$\boldsymbol{\gamma}$ is the penalty term. $\boldsymbol{k = 1, 2, \cdots, K, m = 1, \cdots, N}$.

Gradient boosting is performed on all $\boldsymbol{r_{mk}}$, and the weak regressor function $\boldsymbol{g_k(V, \hat{S}^{(t)})}$ is obtained. It is used to update the regression tree as follows:

$$\boldsymbol{f_k(V, \hat{S}^{(t)}) = f_{k-1}(V, \hat{S}^{(t)}) + v g_k(V, \hat{S}^{(t)})}$$

Loop iterative formulas at the end of step (1) until the iteration converges or the preset number of iteration thresholds are reached. The result is as follows:

$$\boldsymbol{r_t(V, \hat{S}^{(t)}) = f_k(V, \hat{S}^{(t)})}$$

(3) Feature point search and fit

(a) Initialize the face shape vector. It is assumed that there are n face images and their corresponding face shapes $\boldsymbol{S_n}$ in the training dataset $(V_1, S_1), \cdots (V_n, S_n)$, and the average shape of all training images is used as the initial shape for training.

(b) The tree splits. After all the images are input into the first regression tree, the tree is split according to the pixel difference between the key points of the face in each image and the pair of feature points, until the branch cannot reach the leaf node, the split of the current regression tree is completed.

(c) Integrate the residual sum of all regression trees. The leaf node of each regression tree estimator in the weak regressor stores the difference between the current face key point and the actual key point in the training image in this node. After calculating the residuals of all the leaf nodes of the first regression tree, input the residual results calculated by the first regression tree into the second regression tree constructed. If the residual values of all leaf nodes are calculated, the residual results are filled into the third regression tree constructed, and the process is repeated until the end of the cycle.

(d) Repeat steps (b)-(c). The weak regressor iterates many times, and integrates the calculation results of all the weak regressors until enough tree models are established, so that the face shape output by the model is optimal. The process of face key points gradually approaching the actual face shape during training is shown in Figure 3.

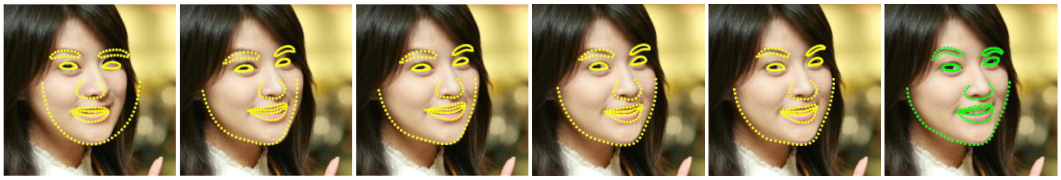

(a) $T = 0$ (b) $T = 1$ (c) $T = 2$ (d) $T = 3$ (e) $T = 10$ (f) Ground truth

Figure 3. Training process of the facial key point

1.3 Head feature extraction algorithm

Considering that the infrared camera is relatively fixed, in order to facilitate the estimation of the feature state of the driver's head, the driver's head is abstracted as an intangible rigid object. The

characteristic of a rigid body is that the relative positions of its internal feature points do not change, and the shape and size of the rigid body itself do not change during motion, and the object itself only performs rotation and translation transformations.

Based on the above characteristics, the driver's head is approximately regarded as a rigid body in this paper. The head pose is calculated based on the PnP algorithm (Perspective n Points) and the Dlib face key point model to calculate the correspondence between the coordinates of 15 2D face key points and the coordinate system in the 3D face head model.

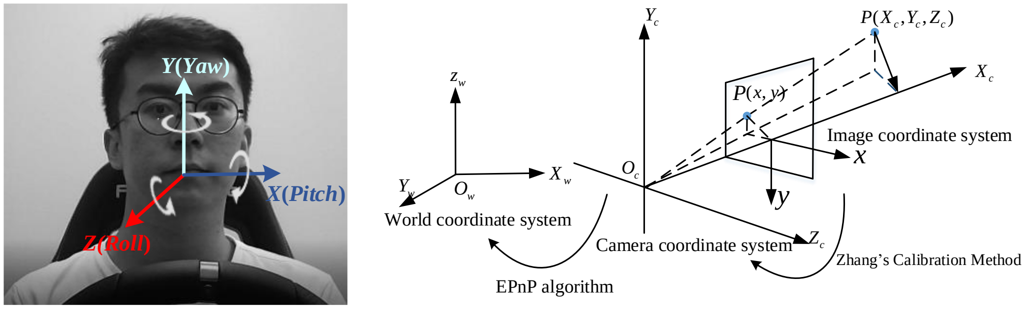

Figure 4. Euler angle diagram.

The EPnP method is used for head pose estimation in this paper, which is based on MTCNN to identify five 2D landmarks on the face. Through the rotation matrix of the camera, the three-dimensional point distribution in the world coordinate system can be mapped to the two-dimensional image feature point distribution, and the rotation matrix can be converted into the Euler angle feature parameters of the head pose. EPnP is a high-precision and fast pose estimation method with strong anti-interference ability. According to the EPnP method, four virtual control points are set in the three dimensional space, denoted as $\boldsymbol{C_1}$, $\boldsymbol{C_2}$, $\boldsymbol{C_3}$ and $\boldsymbol{C_4}$. Label each reference point as a weighted sum of control points as follows:

$$\boldsymbol{P_i^w = \sum_{j=1}^{4} \delta_{ij} c_j^w \,, with \sum_{j=1}^{4} \delta_{ij}}$$

$\boldsymbol{P_i^c}$ is $\boldsymbol{P_i^w}$ mapped to the corresponding point in the camera coordinate system. $\boldsymbol{c_j^c}$ represents the virtual point corresponding to $\boldsymbol{c_j^w}$.

Suppose the projection of the 3D reference point in the pixel coordinate system is $(\boldsymbol{u_i}, \boldsymbol{v_i})$. The formula for calculating $\boldsymbol{P_i^c}$ can be rewritten as follows:

$$z_j^c \begin{bmatrix} \boldsymbol{u_i} \\ \boldsymbol{v_i} \\ 1 \end{bmatrix} = K\boldsymbol{p_i^c} = \boldsymbol{K} \sum_{j=1}^{4} \boldsymbol{\delta_{ij} c_j^c} = \begin{bmatrix} \boldsymbol{f_x} & \boldsymbol{0} & \boldsymbol{u} \\ \boldsymbol{0} & \boldsymbol{f_y} & \boldsymbol{v} \\ \boldsymbol{0} & \boldsymbol{0} & \boldsymbol{1} \end{bmatrix} \sum_{j=1}^{4} \boldsymbol{a_{ij}} \begin{bmatrix} \boldsymbol{x_j^c} \\ \boldsymbol{y_j^c} \\ \boldsymbol{z_j^c} \end{bmatrix}$$

$\boldsymbol{z_j^c}$ is the projection depth of the reference point. $\boldsymbol{K}$ is the camera's internal parameter matrix. $\boldsymbol{u}$ and $\boldsymbol{v}$ are the optical centers of the camera. $\boldsymbol{f_x}$ and $\boldsymbol{f_y}$ are the focal length of the camera.

The correspondence between each reference point and pixel coordinate point is as follows:

$$\begin{cases} \sum_{J=1}^{4} \boldsymbol{\delta_{ij} f_x x_j^c + \delta_{ij}(u - u_i) z_j^c = 0} \\ \sum_{J=1}^{4} \boldsymbol{\delta_{ij} f_y x_j^c + \delta_{ij}(v - u_i) z_j^c = 0} \end{cases}$$

According to the 4 virtual points and reference points in the camera coordinate system, 8 equations can be obtained, denoted as $\boldsymbol{Mx = 0}$. $\boldsymbol{M}$ is a matrix of $\mathbf{8 \times 12}$, which can be expanded into a $\mathbf{12 \times 1}$ column vector $\boldsymbol{c} = [\boldsymbol{c_1^{c^T}}, \boldsymbol{c_2^{c^T}}, \boldsymbol{c_3^{c^T}}, \boldsymbol{c_4^{c^T}}]^{\boldsymbol{T}}$. Since the coordinates of the four reference points are known in the camera coordinate system, the solution of the three-dimensional to two-dimensional PNP problem is converted into a classical three-dimensional to three-dimensional rigid body motion problem, so that the rotation matrix $\boldsymbol{R}$ and the translation matrix $\boldsymbol{t}$ can be obtained. Rewrite the calculated rotation matrix to get the Euler angle characteristic parameters:

$$\boldsymbol{Pitch = arctan\left(-\frac{r_{31}}{\sqrt{r_{32}^2 + r_{33}^2}}\right), Yaw = arctan\left(\frac{r_{32}}{r_{33}}\right), Roll = arctan\left(\frac{r_{21}}{r_{11}}\right)}$$

The pseudocode description based on Euler angle feature parameter extraction is shown in Table 1.

Table 1. The pseudocode description based on Euler angle feature parameter extraction.

Input： The yaw angle list is *Yaw_l*; the pitch angle list is *Pitch_l*; the roll angle list is *Roll_l*; the image coordinate matrix of the selected 15 key points is i*mage_points*; the coordinate matrix of the selected 15 3D key points is *model_points*; the video frame image is *frame*.

function Data_write(*Yaw_l*, *Pitch_l*, *Roll_l*)
Convert *Yaw_l, Pitch_l, Pitch_l* list to dimension
Euler angle ← np.hstack((*Yaw_l, Pitch_l, Pitch_l*))
data ← pd.DataFrame(*Euler angle*)
Use the data.to_csv( ) function to save the Euler angle parameters as a csv file
end function
Using Zhang's calibration method to obtain camera internal parameters *camera_matrix*, distortion coefficient *dist_coeffs*
Calculate the rotation matrix *rotation_vector* ← cv2.solvePnP(*model_points*, *image_points*, *camera_matrix*, *dist_coeffs*)
while cap.isOpened() == True **do**
Use the cv2.resize() function to convert the *frame* size of the input video image
Convert *rotation_vector* to Euler angle parameters
    try：
        if len(*Yaw_l*) = len(*Pitch_l*) = len(*Roll_l*) **do**
            Data_write(*Yaw_l*, *Pitch_l*, *Roll_l*)
    except：
        break

end while
Output: Euler angle feature parameters in csv file format

2 Supplementary Figures and Tables

2.1 Supplementary Figures

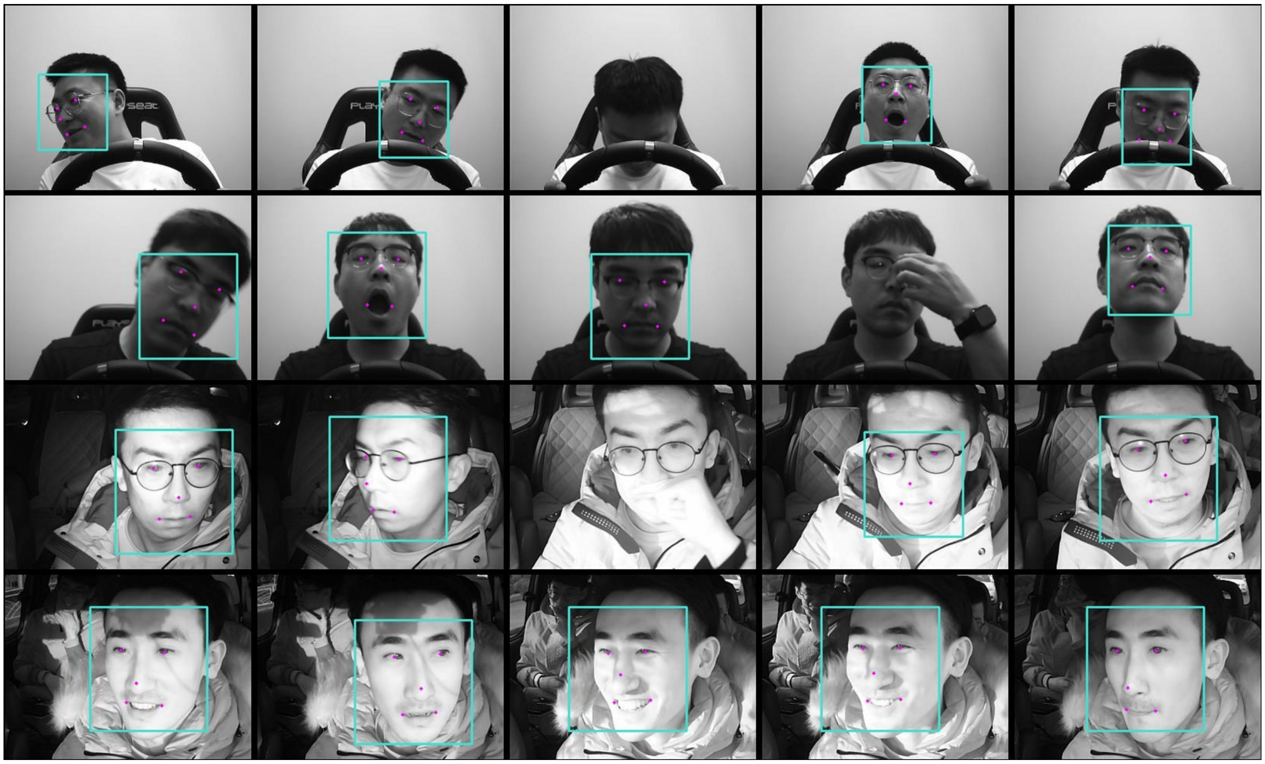

Supplementary Figure 1. The face detection effect of improved MTCNN.

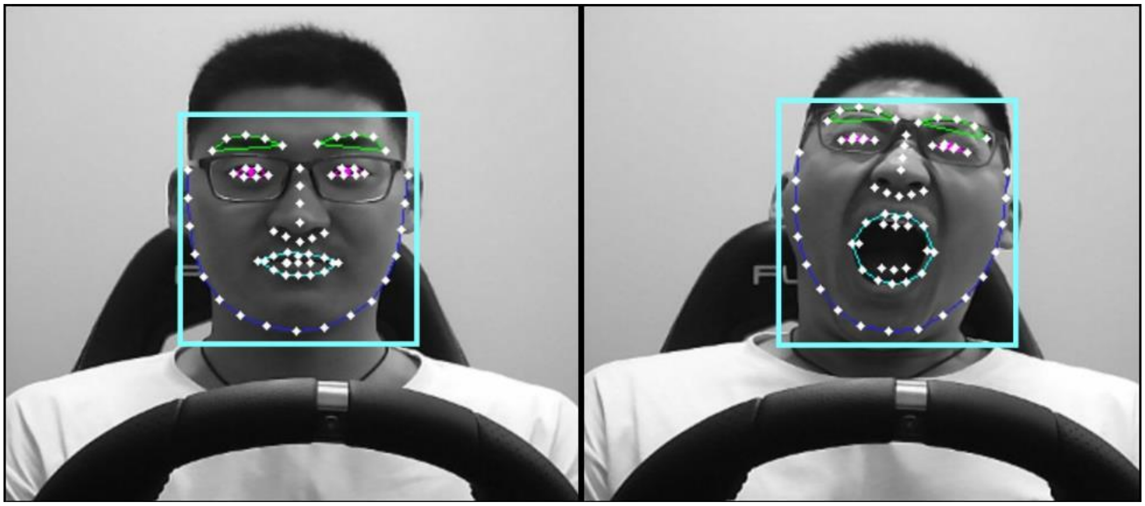

Supplementary Figure 2. The key point positioning effect of Dlib.

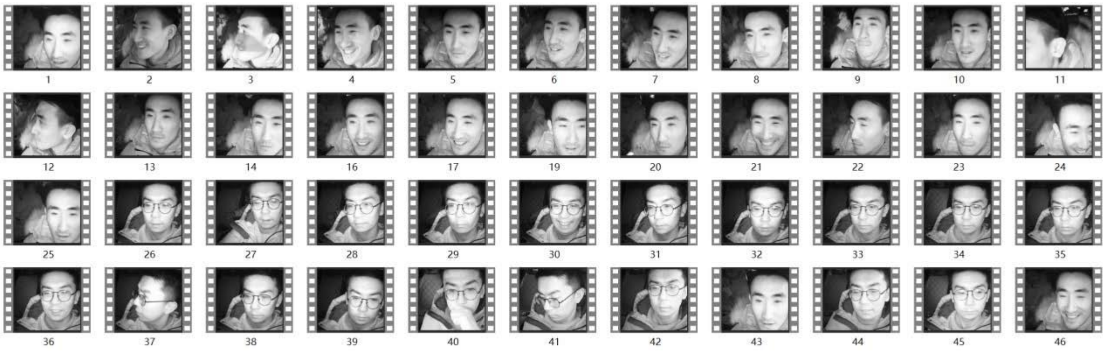

Supplementary Figure 3. Part of the experimental data.

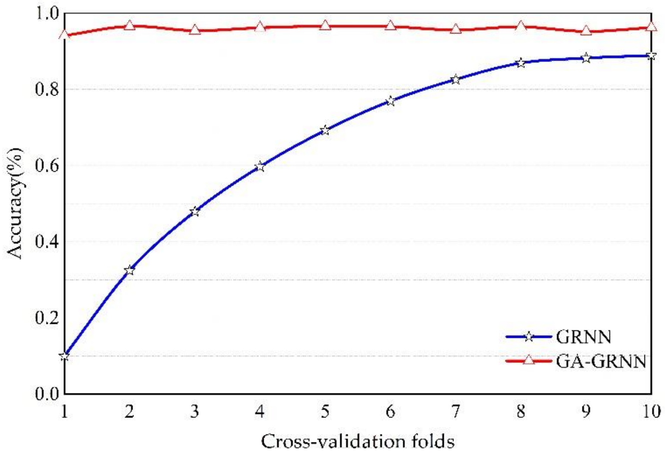

Supplementary Figure 4. GRNN identification accuracy before and after optimization.

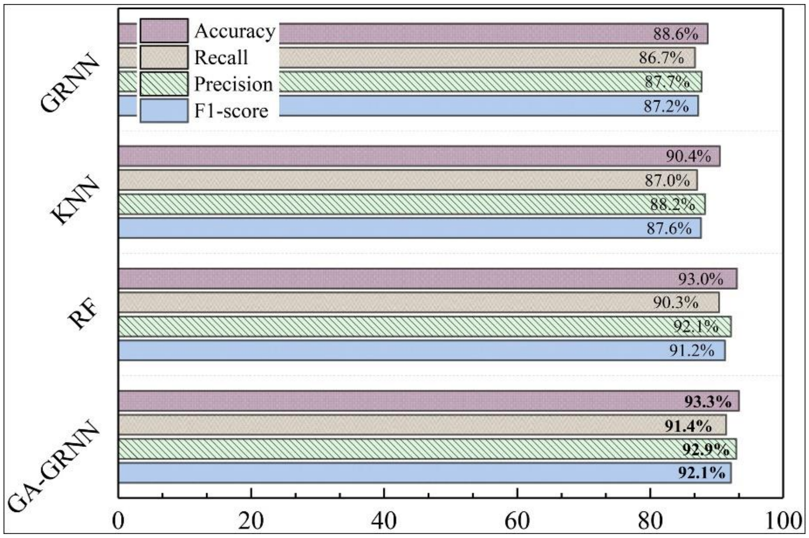

Supplementary Figure 5. Validation results.

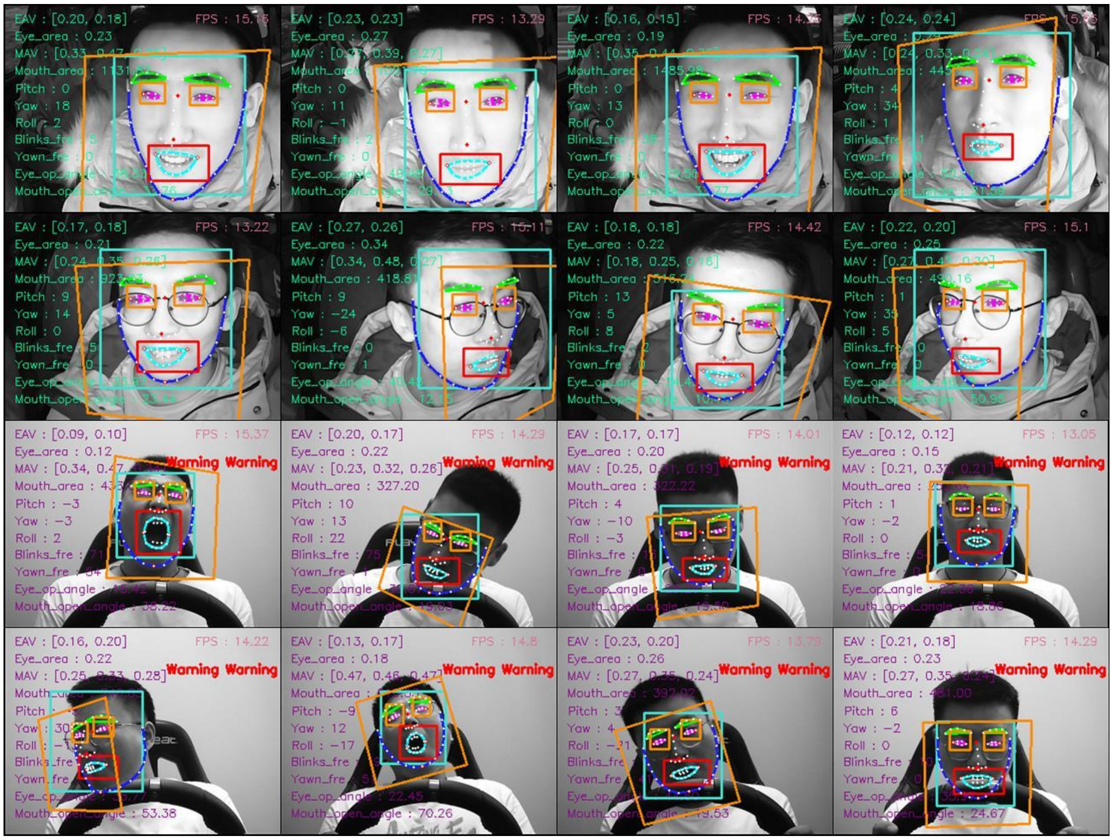

Supplementary Figure 6. Validation results example.

2.2 Supplementary Tables

Supplementary Table 2. The basic information of the participants.

Number	Gender	Age	Driving age	Wear glasses
1	Male	25	3	Yes
2	Male	25	1	No
3	Male	28	5	No
4	Female	36	9	No
5	Male	51	15	Yes
6	Female	26	4	No
7	Male	26	2	No
…	…	…	…	…
33	Male	39	10	No

Supplementary Table 2. Two category KSS scale.

Fatigue level	Fatigue degree	Status description	Driving status
1	Extremely awake	Responsive.	Normal driving
2	Very awake	Clear thinking.	
3	Wide awake	Can control the vehicle stably.	
4	Comparatively awake	Decreased driving performance.	Fatigue driving
5	Neither awake nor sleepy	Can't concentrate.	
6	A little sleepy	Fatigue symptoms are obvious.	
7	Sleepy but easy to stay awake	Frequent yawning and nodding.	

8	Sleepy but struggling to stay awake	Close the eyes for a long time.
9	Severe drowsiness that requires effort to overcome	

Supplementary Table 3. Comparison results of the face detection model.

Algorithm	Accuracy(%)	Detection speed($ms \cdot f^{-1}$)
Harr + Adaboost	88.2	39
Hog + SVM	91.6	43
MTCNN	93.4	35
SSD [49]	97.7	36
Improved MTCNN	95.3	34

Supplementary Table 4. Driver fatigue characterization parameter information.

No		Symbol	Parameter type	Unit	Description
1	X_1	F_{blink}	Blink rate	-	Number of times the driver blinks in a fixed period of time.
2	X_2	EOA	Canthus opening degree	deg	Angle at which the corner of the driver's eyes changes.
3	X_3	EAV	Vertical and horizontal dimensions of the eye	-	Degree to which the driver's eyelid distance varies.
4	X_4	S_{ar}	Closed eye area	px^2	Degree to which the driver's eyes are closed.
5	X_5	F_{yawn}	Yawn frequency	-	Number of times the driver yawned in a fixed period of time.
6	X_6	MAV	Mouth longitudinal dimension	-	Degree of variation in the distance between the driver's lips.
7	X_7	MOA	Mouth opening degree		Angle at which the corners of the driver's mouth change.
8	X_8	S_{mp}	Mouth closed area	px^2	Degree to which the driver's mouth opens.
9	X_9	Pitch	Pitch angle	deg	Changing angle of the driver's head about the x-axis.
10	X_{10}	Yaw	Yaw angle	deg	Changing angle of the driver's head about the y-axis.
11	X_{11}	Roll	Roll angle	deg	Changing angle of the driver's head about the z-axis.
