# Supplementary figures and images for: A real-time driver fatigue identification method based on GA-GRNN
Source: Front Public Health. 2022 Oct 20;10:991350. doi: 10.3389/fpubh.2022.991350 (PMC9632354; doi:10.3389/fpubh.2022.991350)

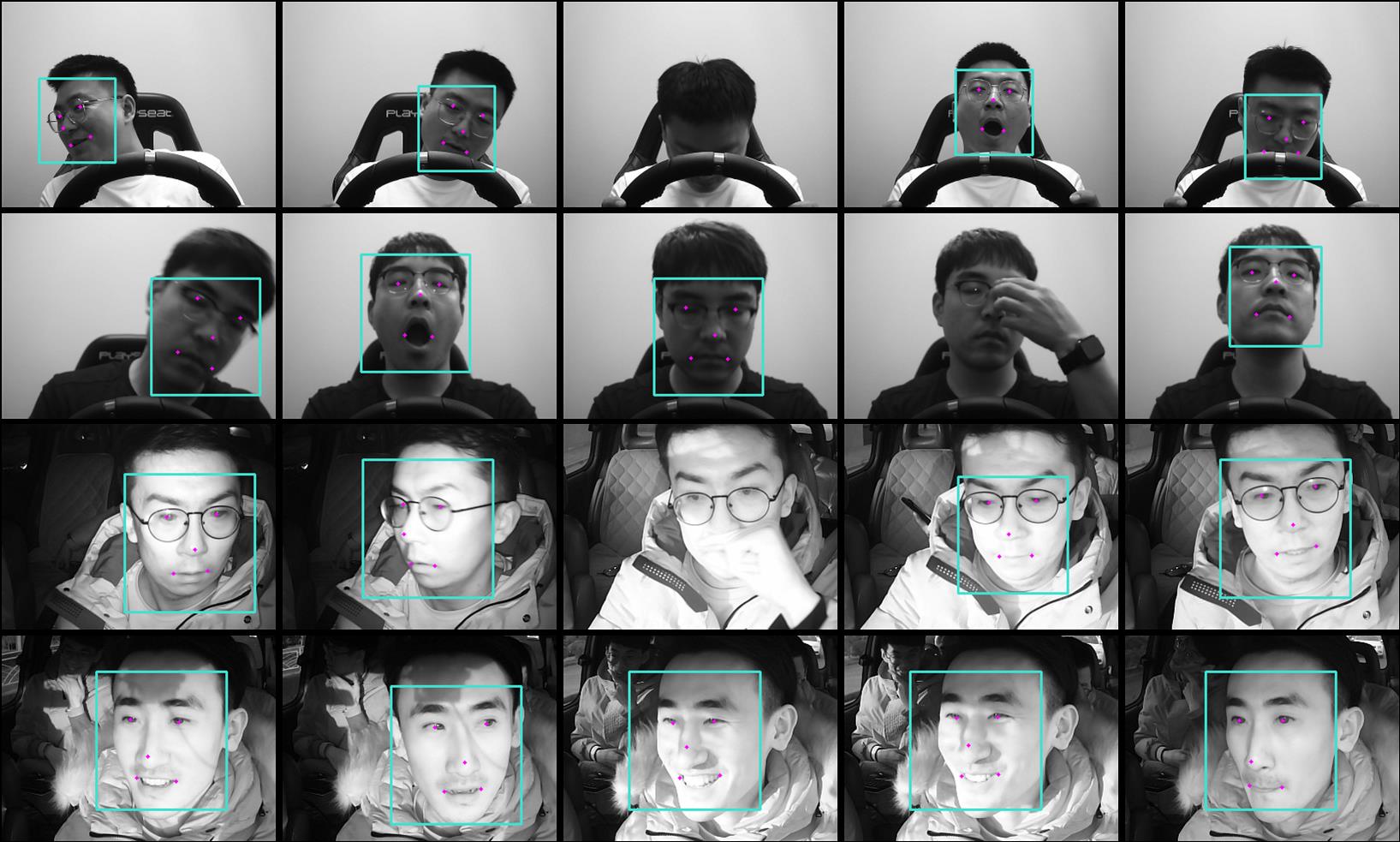

Supplement: Supplementary file 1 [file Data_Sheet_1.ZIP › Supplementary_Figures/Supplementary Figure 1. The face detection effect of improved MTCNN..jpg]

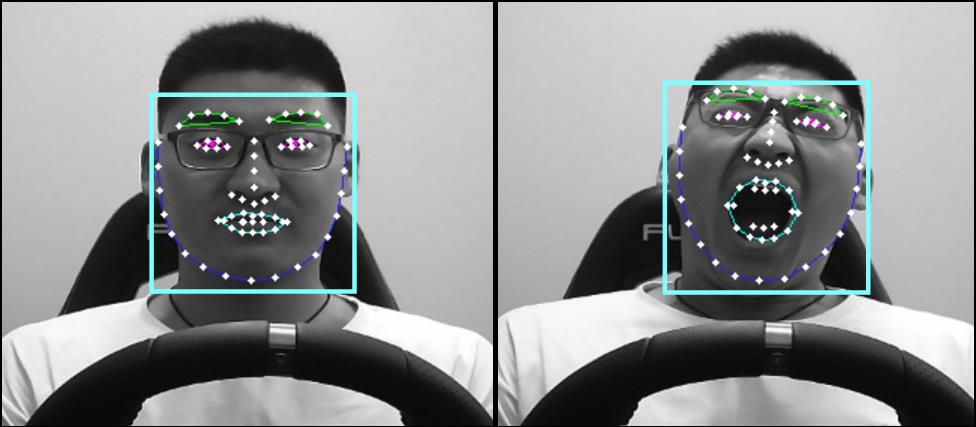

Supplement: Supplementary file 1 [file Data_Sheet_1.ZIP › Supplementary_Figures/Supplementary Figure 2. The key point positioning effect of Dlib..jpg]

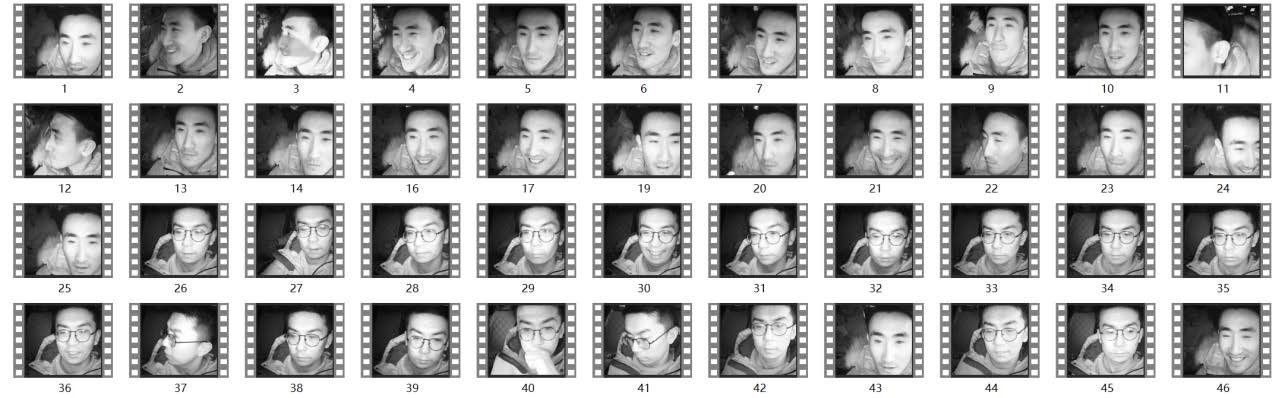

Supplement: Supplementary file 1 [file Data_Sheet_1.ZIP › Supplementary_Figures/Supplementary Figure 3. Part of the experimental data..jpg]

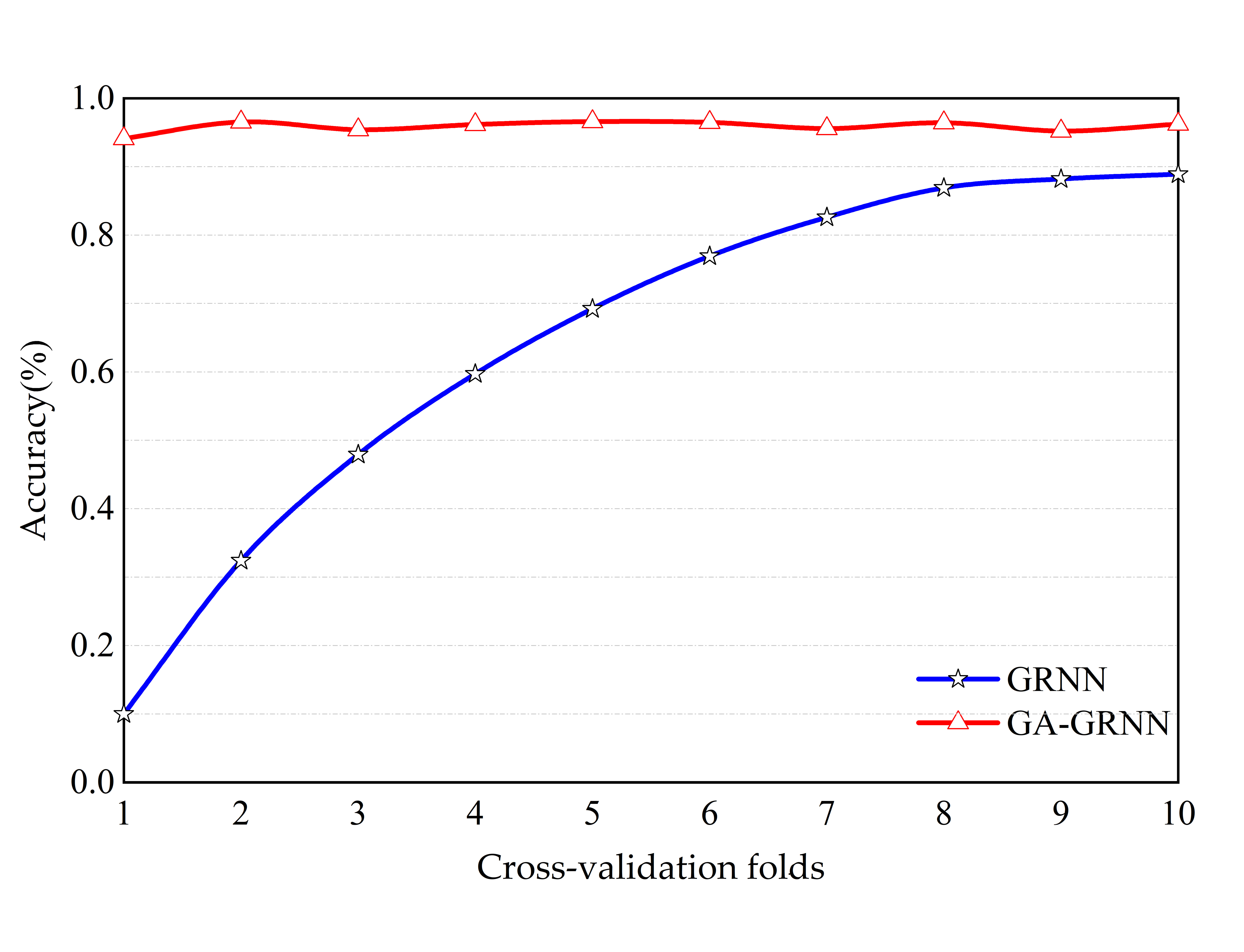

Supplement: Supplementary file 1 [file Data_Sheet_1.ZIP › Supplementary_Figures/Supplementary Figure 4. GRNN identification accuracy before and after optimization..jpg]

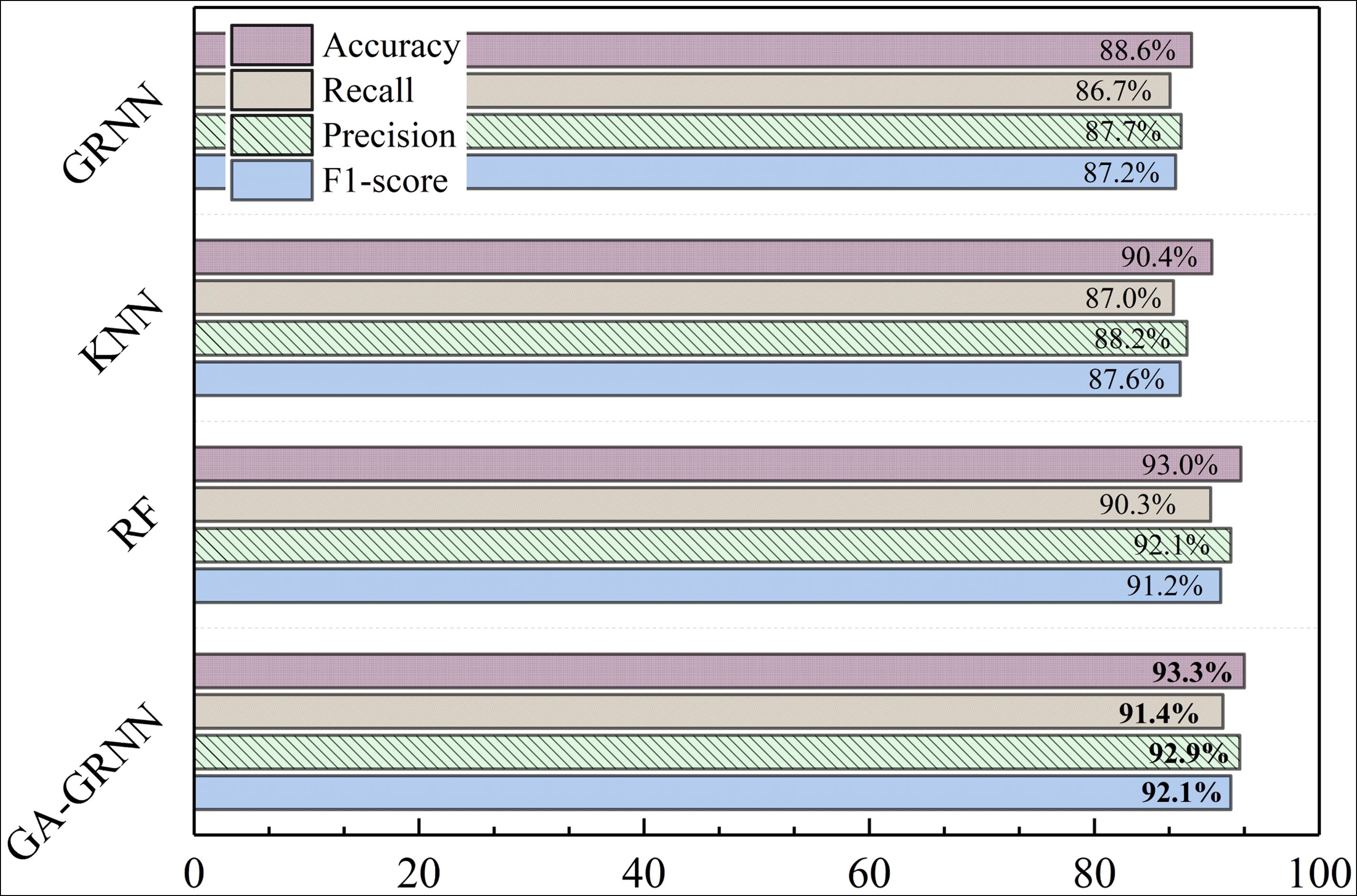

Supplement: Supplementary file 1 [file Data_Sheet_1.ZIP › Supplementary_Figures/Supplementary Figure 5. Validation results..jpg]

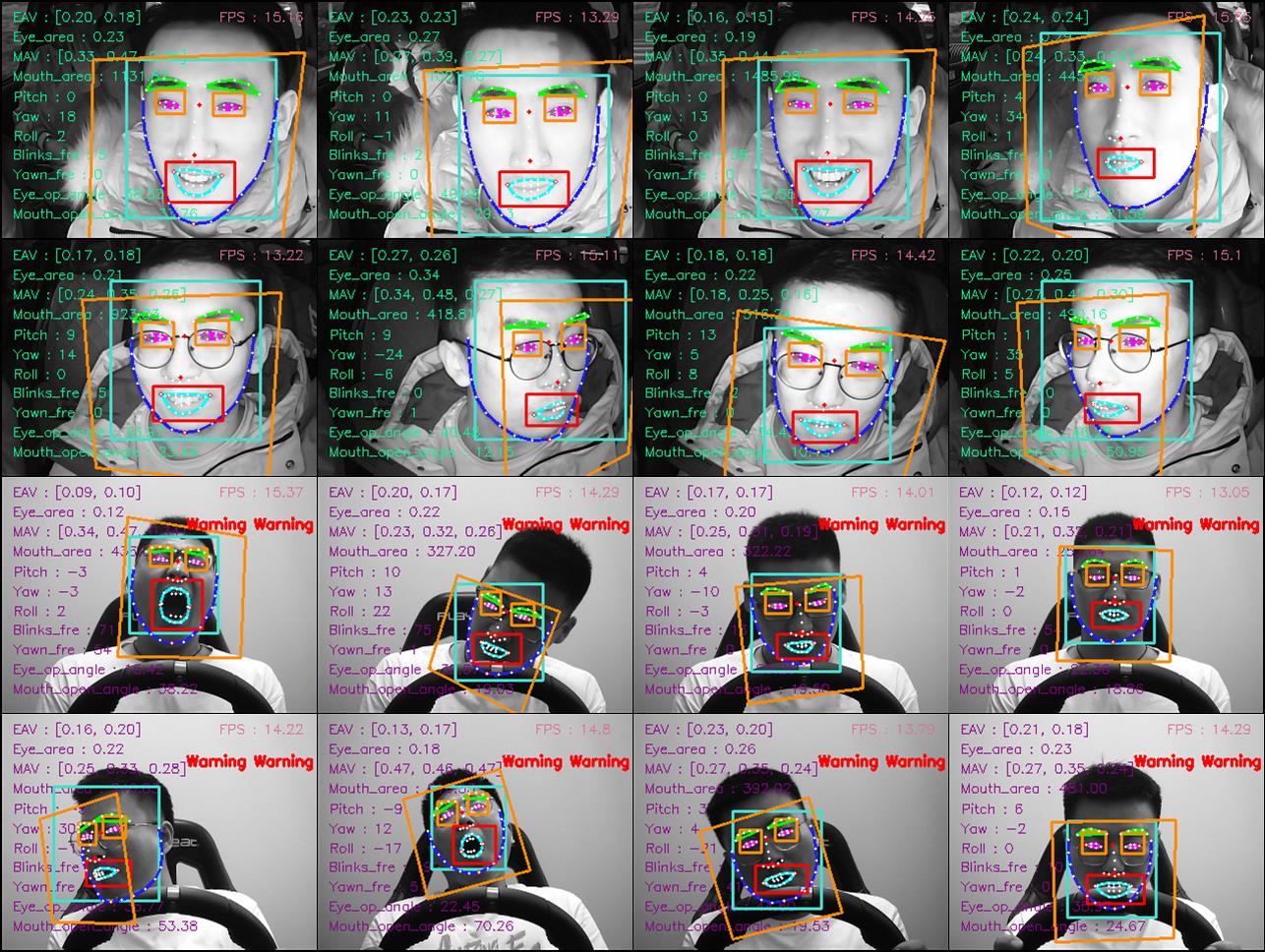

Supplement: Supplementary file 1 [file Data_Sheet_1.ZIP › Supplementary_Figures/Supplementary Figure 6. Validation results example..jpg]
